# Neuroanatomical Changes in the Stopping Network Across the Adult Lifespan Assessed With Quantitative and Diffusion MRI

**DOI:** 10.1002/hbm.70240

**Published:** 2025-06-05

**Authors:** Sarah A. Kemp, Pierre‐Louis Bazin, Steven Miletić, Russell J. Boag, Max C. Keuken, Mark R. Hinder, Birte U. Forstmann

**Affiliations:** ^1^ Integrative Model‐Based Cognitive Neuroscience Research Unit, Department of Psychology University of Amsterdam Amsterdam North Holland the Netherlands; ^2^ Sensorimotor Neuroscience and Ageing Research Laboratory, School of Psychological Sciences, College of Health and Medicine University of Tasmania Hobart Tasmania Australia; ^3^ Full Brain Picture Analytics Leiden South Holland the Netherlands; ^4^ Department of Psychology, Faculty of Social Sciences Leiden University Leiden South Holland the Netherlands

**Keywords:** ageing, DWI, iron, myelin, qMRI, response inhibition, white matter

## Abstract

Response inhibition, the cancellation of planned movement, is essential for everyday motor control. Extensive fMRI and brain stimulation research provides evidence for the crucial role of a number of cortical and subcortical regions in response inhibition, including the subthalamic nucleus (STN), presupplementary motor area (preSMA) and the inferior frontal gyrus (IFG). Current models assume that these regions operate as a network, with action cancellation originating in the cortical areas and then executed rapidly via the subcortex. Response inhibition slows in older age, a change that has been attributed to deterioration or changes in the connectivity and integrity of this network. However, previous research has mainly used whole‐brain approaches when investigating changes in structural connectivity across the lifespan or has used simpler measures to investigate structural ageing. Here, we used high‐resolution quantitative and diffusion MRI to extensively examine the anatomical changes that occur in this network across the lifespan. We found age‐related changes in iron concentration in these tracts, increases in the apparent diffusion coefficient and some evidence for a decrease in myelin content. Conversely, we found very little evidence for age‐related anatomical changes in the regions themselves. We propose that some of the functional changes observed in these regions in older adult populations (e.g., increased BOLD recruitment) are a reflection of alterations to the connectivity between the regions rather than localised regional change.

## Graphical abstract

Response inhibition enables us to modify ongoing motor actions, but this process becomes impaired in older age. We examined age‐related anatomical changes to the response inhibition network using diffusion and quantitative MRI, and found that white matter degradation and iron accumulation may drive these observed behavioural changes.

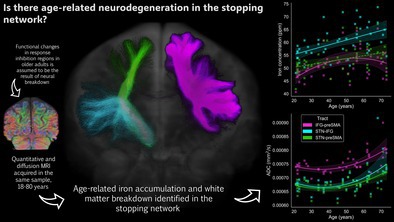

**Summary**

- Response inhibition relies on a specific cortical–subcortical network including the presupplementary motor area, inferior frontal gyrus and subthalamic nucleus. The functionality of this network changes with age; we examined how structural changes may underpin this changed functionality.
- Age‐related changes were more pronounced in the connections between these regions, rather than the regions themselves. Findings showed increased iron concentration, higher diffusivity and potential decrease in myelination of the white matter tracts.
- The functional changes seen in older adults (e.g., increased BOLD activation) may reflect compensatory recruitment due to degraded connectivity, rather than direct deterioration of individual brain regions.

## 1 Introduction

Response inhibition is an essential part of everyday motor control, and is crucial for successfully navigating dynamic and complex environments. An important part of response inhibition is action cancellation, the sudden termination of planned or already‐initiated movement (Sebastian, Pohl, et al. 2013; Wessel and Aron 2017). Action cancellation is impeded in older age, with older adults typically showing delayed stopping latencies (Rey‐Mermet and Gade 2018; Smittenaar et al. 2013; Tsvetanov et al. 2018). A consistent finding across the literature is that the subthalamic nucleus (STN), inferior frontal gyrus (IFG) and presupplementary motor area (preSMA) play central roles in implementing action cancellation (see, e.g., Aron et al. 2007; Diesburg and Wessel 2021; Isherwood et al. 2025; Isherwood et al. 2021; Lee et al. 2016; Lofredi et al. 2021; Xu et al. 2016; Zandbelt et al. 2013). These regions are thought to form a core fronto‐basal ganglia ‘stopping network’, which rapidly interrupts motor activity when required. Importantly, age‐related decline in action cancellation has been linked to functional and structural changes within this network, including reduced engagement of prefrontal and basal ganglia regions, shifts in inhibitory control strategies and reduced structural connectivity between the STN, preSMA and IFG (Bissett and Logan 2011; Bloemendaal et al. 2016; Coxon et al. 2012; Van De Laar et al. 2011). However, the specific neural breakdown that occurs in these cortical and subcortical regions is incompletely understood. Here, we combine multiple imaging approaches to extensively examine the anatomical changes that occur in this network across the adult lifespan.

The neural correlates of response inhibition and action cancellation are highly complex, and can vary depending on the specificities of the task (Isherwood et al. 2023; Puri et al. 2018; Sebastian, Pohl, et al. 2013). Current models of response inhibition propose that these regions form a complex ‘stopping network’, and regulate the initiation and cancellation of actions through three cortico‐basal ganglia pathways (the direct, indirect and hyperdirect pathways), which send commands to the effector muscles via the STN and other parts of basal ganglia (Graybiel 2000; Rocha et al. 2023; Schroll and Hamker 2013). This framework was initially developed from research involving rodents and nonhuman primates (see, e.g., Coudé et al. 2018; Eagle et al. 2008; Nambu et al. 2002; Nougaret et al. 2013; Schmidt et al. 2009), but the anatomical plausibility of the hyperdirect pathway, which sends ultra‐fast signals between the STN and the prefrontal cortex, has recently been established in humans (Bingham et al. 2023; Chen et al. 2020; Narayanan et al. 2020). Action cancellation itself was originally thought to be implemented via the hyperdirect pathway, originating in the IFG (Aron et al. 2004, 2014; Aron and Poldrack 2006; Bingham et al. 2023; Cavanagh et al. 2014), while two‐stage models of action cancellation hypothesise that both the indirect and hyperdirect pathways are critically involved in action cancellation, originating in humans in the preSMA and IFG respectively (Diesburg and Wessel 2021; Frank 2006; Schmidt and Berke 2017). As well as their individual contributions to action cancellation, the structural connectivity of these three regions has been shown to be an important predictor of stopping performance and other measures of response inhibition (Boen et al. 2022; Forstmann et al. 2012; King et al. 2012; Singh et al. 2021). This highlights the importance of considering how these regions cooperate and interact as a network in order to effectively coordinate movement, as well as considering their individual roles.

Action cancellation is frequently investigated using the stop‐signal task (SST), where participants have to make a default ‘go’ response in every trial, occasionally needing to cancel or inhibit this response after it has been initiated. Performance in trials where the response is cancelled is normally quantified by the stop‐signal reaction time (SSRT), which estimates the speed of the latent stop process (Verbruggen et al. 2019). Older adults generally exhibit slower response times in SSTs and other response inhibition tasks (see e.g., Healey et al. 2024; Kang et al. 2022; Nikitenko et al. 2020; Rey‐Mermet and Gade 2018), but also show notably longer SSRTs compared to younger adults (Hsieh and Lin 2017; Van De Laar et al. 2011; Williams et al. 1999). Importantly, this increase in SSRT cannot solely be attributed to the aforementioned general slowing of response times (Bedard et al. 2002; Hu et al. 2018), indicating there is neural degradation unique to the stopping process in older age.

Alongside these behavioural changes, older adults also show changed neural recruitment patterns in response inhibition tasks. During SSTs, older adults tend to show less activation in the traditional ‘stopping regions’ (STN, preSMA, IFG), but increased activity in a wide range of additional regions (Hu et al. 2018; Hu et al. 2019; Kang et al. 2022; Kleerekooper et al. 2016; Sebastian, Baldermann, et al. 2013). These broader recruitment patterns have also been observed in other tasks (see e.g., Kennedy et al. 2015; Zhu et al. 2010). Theoretical frameworks such as the dedifferentiation hypothesis, Scaffolding Theory of Ageing and Cognition (STAC) and Compensation‐Related Utilisation of Neural Circuits Hypothesis (CRUNCH) postulate that these broader recruitment patterns serve as a compensatory mechanism for localised deterioration in the regions that were previously more specialised in performing these tasks (Geerligs et al. 2015; Kang et al. 2022; Reuter‐Lorenz and Park 2014). This more widespread recruitment serves to maintain behavioural performance, but as task demand increases or when there is further structural degradation, this compensation is less effective and behavioural performance begins to decline. Age‐related structural decline in the brain has indeed been observed, but patterns of decline vary from region to region and between different networks (Geerligs et al. 2015; Zimmermann et al. 2016). Changes in specific networks and regions need therefore to be individually considered and cannot be described in a generalised manner.

Age‐related connectivity changes have previously been investigated using diffusion‐weighted imaging (DWI), a structural application of magnetic resonance imaging (MRI) which quantifies the movement of water molecules in the brain. This movement is anatomically constrained by brain structures such as white matter and fibre bundles. Determining the magnitude and direction of water diffusion thus enables estimation of the underlying anatomy (Grier 2020; O'Donnell and Westin 2011). There are a number of common diffusion metrics, but two widely‐used ones are the apparent diffusion coefficient (ADC) and fractional anisotropy (FA) (Beck et al. 2021; Lazari and Lipp 2021; Porcu et al. 2021; Sullivan and Pfefferbaum 2006). The ADC relates to the net diffusion in a voxel, and will increase as water movement becomes less anatomically constrained (e.g., with breakdown of brain structure). FA is a ratio measure that quantifies the extent to which the water movement is anisotropic (nonuniform). Higher values of FA (closer to 1) indicate that the movement is more constrained. Both measures are generally thought to reflect white matter integrity and be influenced by biophysical measures (Grier 2020; O'Donnell and Westin 2011). These measures have frequently been used to investigate neuroanatomical changes across the adult lifespan (see e.g., Beck et al. 2021; Henriques et al. 2023; Lebel et al. 2012). The changes that occur in these metrics in older age vary from region to region, but generally, FA will increase and ADC will decrease.

Connectivity between the canonical stopping regions (STN, IFG, preSMA), indexed via various diffusion metrics, has been associated with response inhibition performance and SSRT in children, adolescents and young adults (Aron 2007; Boen et al. 2022; King et al. 2012; Madsen et al. 2020; Zhang and Iwaki 2020). While there has been less investigation of older adults, broader changes in diffusion indices (i.e., not specific to the stopping network) have been linked to response inhibition performance (Yang et al. 2019) and connectivity between the preSMA and STN has been found to be predictive of SSRT (Coxon et al. 2012). Aside from these few examples, investigations regarding the anatomical changes that occur in this network across the adult lifespan have been limited, particularly in healthy populations.

Further, although the aforementioned diffusion metrics are often assumed to directly index myelin integrity, they are likely influenced by a range of microstructural properties in older adults, including axonal loss, fibre dispersion and variability in fibre and neurite orientation (Beck et al. 2021; Grussu et al. 2017; Lazari and Lipp 2021), and are thus relatively unspecific in terms of the anatomical changes they capture. Assessments of neuroanatomical ageing can therefore be complemented by other imaging techniques. Quantitative MRI (qMRI) can be used to estimate histological measures of brain tissue, such as iron and myelin, in vivo (Keuken et al. 2017; Madden and Merenstein 2023; Miletić et al. 2022; Weiskopf et al. 2015). As well as being critical for normal brain function, these biophysical properties are highly relevant in investigations of the ageing brain. Demyelination is a hallmark of ageing, and iron is increasingly recognised as an important factor in both diseased and healthy ageing contexts (Buyanova and Arsalidou 2021). Importantly, age‐related changes in iron and myelin concentrations tend to be region‐specific, with certain areas exhibiting high levels of iron accumulation and others showing minimal changes (Daugherty and Raz 2015; Hagemeier et al. 2012; Miletić et al. 2022). Taken together, assessment and interpretation of neuroanatomical changes across the lifespan need to be quite region‐specific, that is, generalised hypotheses may not apply to individual networks or regions (Geerligs et al. 2015). Utilisation of multimodal imaging approaches aids in building a richer picture of anatomical change, enhancing understanding of the intricate and dynamic changes that occur across the adult lifespan (Tardif et al. 2016).

Here, we combined high‐resolution DWI and ultra‐high field qMRI (acquired at 3 and 7 Tesla respectively) to examine the physiology of the stopping network across the lifespan in an adult sample. We quantified age‐related changes in these measures, providing a comprehensive assessment of the neuroanatomical changes that may precipitate the behavioural changes in action cancellation observed in older populations. Additionally, given that diffusion measures are so frequently assumed to index myelination and the limited availability of DWI and qMRI datasets in the same sample, we explored the relationship between the diffusion measures and age, iron and myelin to guide the interpretation of DWI metrics in future studies.

## 2 Methods

### 2.1 Participants

**FIGURE 1:**
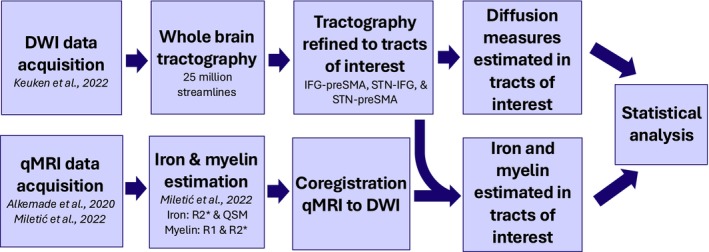
Image acquisition and processing steps. Participants were scanned on two different days, with the qMRI images acquired in the first session and the DWI images in the second. All processing was initially conducted in individual space and then ultimately co‐registered to MNI space.

We used the Amsterdam ultra‐high field adult lifespan database (AHEAD; Alkemade et al. 2020), which comprises multimodal MRI data from 105 healthy adults. We used the subset of these (*N* = 49, 27 females) for whom DWI data are also available (Keuken et al. 2022). Participants were scanned on two different days, some weeks apart. The quantitative images were acquired in the first session and the diffusion images were acquired in the second. The overarching procedure is depicted in Figure 1. Participants were 21–83 years old. Exclusion criteria were any factors that may interfere with MRI scanning, for example, MRI incompatibility, pregnancy, or self‐reported claustrophobia. All procedures were in accordance with the Code of Ethics of the World Medical Association and approved by the Institutional Review Board at the University of Amsterdam (ERB number 2016‐DP‐6897). Informed consent was obtained from participants including permission for the future release of de‐identified data.

### 2.2 DWI

#### 2.2.1 Data Acquisition

The diffusion images were acquired at the Spinoza Centre for Neuroimaging in Amsterdam, the Netherlands with a Philips 3 T Ingenia CX MRI scanner using a 32‐channel receiver head coil. We used an MS‐ME (multi slice spin‐echo) single shot sequence with 1.28 mm isotropic resolution. Diffusion weighting was isotopically distributed along 48, 56 and 64 directions with *b* values of 700 s/mm^2^, 1000 s/mm^2^ and 1600 s/mm^2^ respectively. Due to memory limitations, the 64‐direction scan was split into two 32‐direction scans. Twelve b_0_ volumes were acquired within the MS‐ME sequences and an additional eight were acquired at the end with an inverted fat shift direction. Scan parameters: TE = 78 ms, FA = 90°, TR = 8100 ms, acceleration factor SENSE_AP_ = 2, FOV = 205 × 205, slice gap = 0 mm, TA_b0_ = 00:49 min, TA_b700_ = 13:48 min, TA_b1000_ = 17:19 min, TA_b1600_ = 9:44 min for each half, that is, 19:28 min in total. Total acquisition time ≈100 min.

#### 2.2.2 Preprocessing

The preprocessing pipeline for this diffusion dataset has been previously reported (Keuken et al. 2022). In brief, all data were denoised using the PCA‐based function *dwidenoise* in MRtrix V3.0.2 (Tournier et al. 2019). Gibbs artefacts were removed using the *mrdegibbs* function in MRtrix, and susceptibility‐induced off‐resonance field distortions were corrected using the different phase‐encoding blips of the b0 images in *topup*, implemented with FSL V5.0.11 (Smith et al. 2004). The output from *topup* was used to apply eddy current correction using *eddyOpenMP* in FSL. Finally, an N4 bias correction was applied using *dwibiascorrect* and a brain mask estimated using *dwi2mask*, both with MRtrix.

#### 2.2.3 Tractography

Connectivity between the three ROIs (IFG‐preSMA, IFG‐STN and STN‐preSMA in each hemisphere) was estimated from whole‐brain tractography, implemented using MRtrix (Tournier et al. 2019). This process was performed in individual space for each participant. The final six tracts were transformed into MNI space via 2‐step registration using ANTS (SyN for Step 1 and SyNAggro for Step 2).

From the preprocessed diffusion images (see 2.2.2), fibre orientation distributions (FODs) were estimated using *dwi2fod* (Tournier et al. 2004). We used the *msmt_csd* algorithm, which is designed for data with multiple *b* values (Jeurissen et al. 2014). We then performed whole‐brain tractography based on the FODs using *tckgen* with 25 million streamlines. We used the iFOD2 (Second‐Order Integration Over FODs) algorithm, a probabilistic algorithm that is able to track fibres through regions with high levels of fibre crossing and complex geometry such as the subcortex (Tournier et al. 2010). Note that we adjusted the FOD amplitude cutoff point for track termination from 0.1 to 0.05; we found the original cutoff value to be too conservative for some of the older adults in the dataset, drastically limiting the number of streamlines identified for those participants in regions with white matter inflammation or lesions.

**FIGURE 2:**
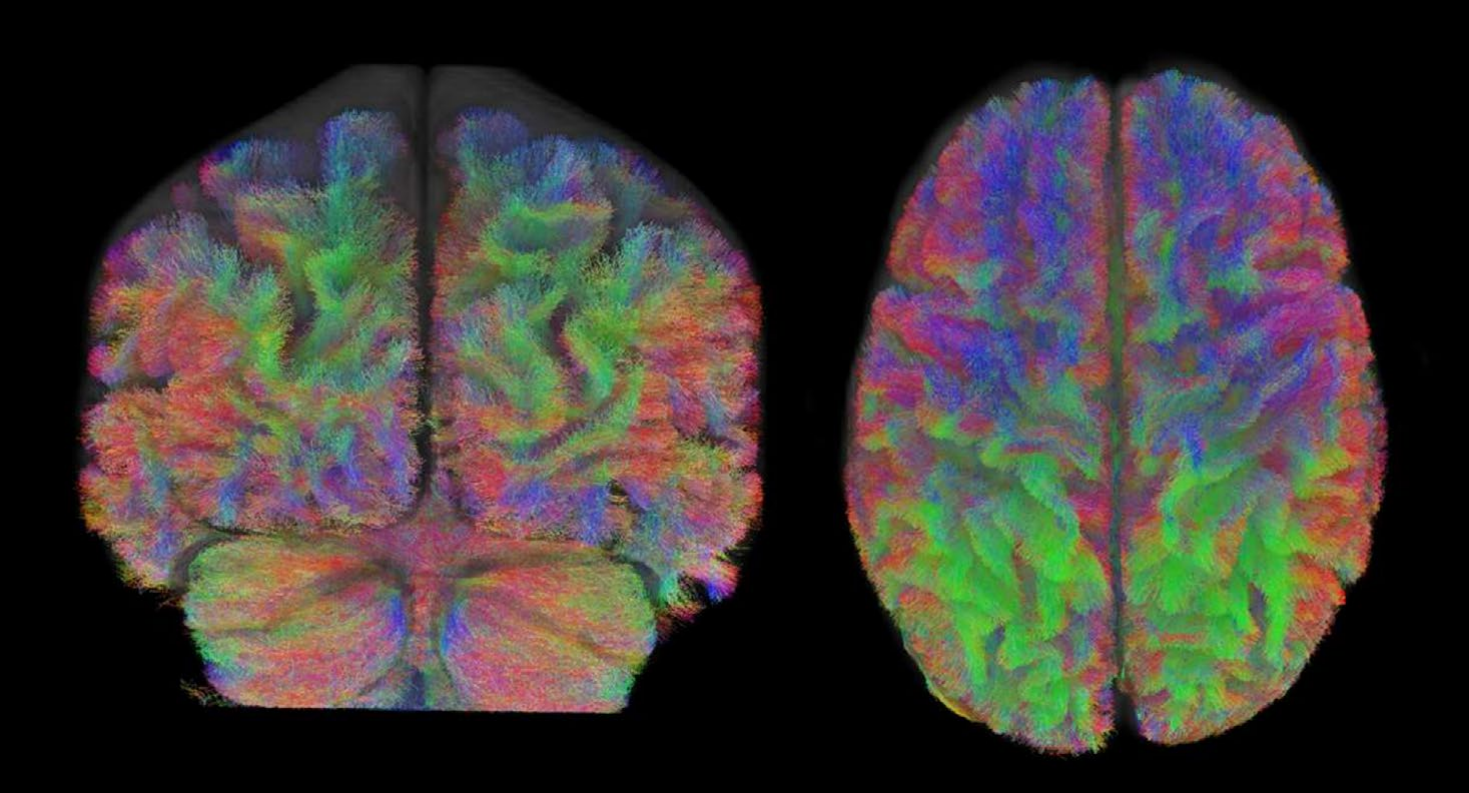
Whole‐brain tractograms from two separate participants, shown in the coronal (left) and axial (right) planes. Note that these images show subsampled tractograms, with 200,000 streamlines for ease of visualisation. The tractography used for analysis had 25 million streamlines, estimated using fibre orientation distributions. Colours represent the local orientation of that tract (red: Left–right; green: Anterior–posterior; blue: Inferior–superior).

We then refined the whole‐brain tractograms using *tcksift2* (Smith et al. 2015) and isolated the streamlines that were connected to any of the ROIs using *tck2connectome* (Hagmann et al. 2008). The STN was defined based on the Multi‐Contrast Anatomical Subcortical Structures Parcellation (MASSP) automated algorithm (Bazin et al. 2020). The IFG and preSMA were defined based on masks from Neubert and colleagues (IFG: Neubert et al. 2014; preSMA: Neubert et al. 2015). Subsampled tractograms from two participants are shown in Figure 2.

**FIGURE 3:**
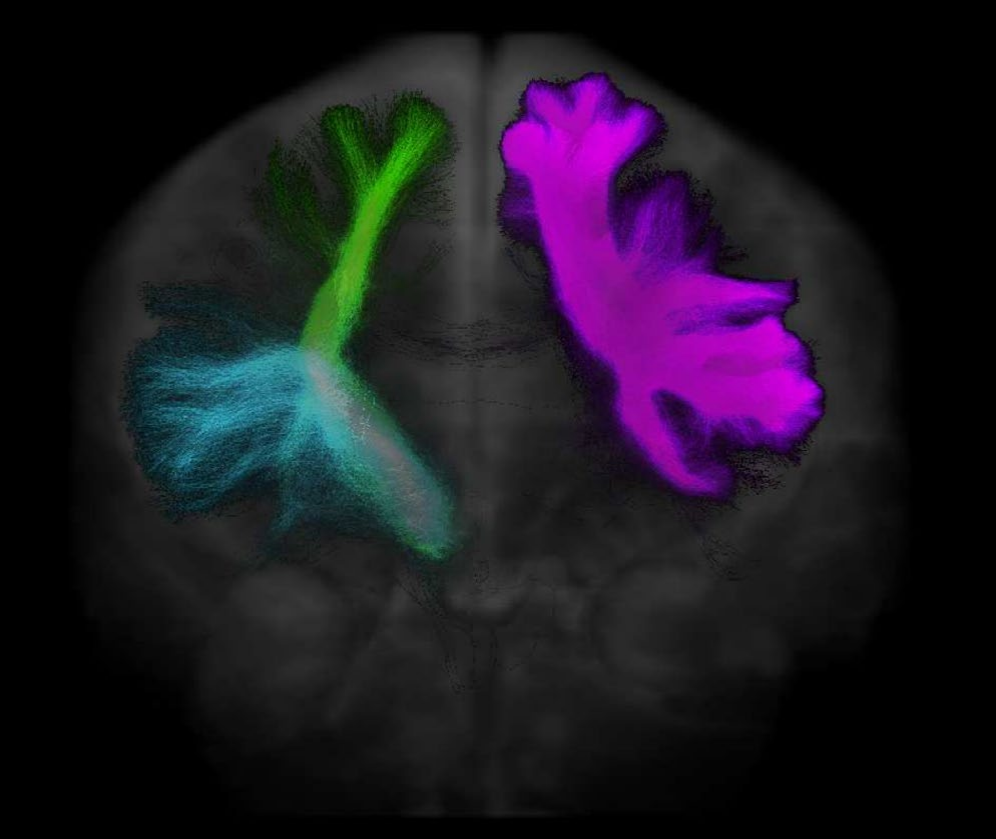
Three of the six tracts of interest from one participant. The tracts of interest were the STN‐IFG, STN‐preSMA and IFG‐preSMA in each hemisphere, but for ease of visualisation, each tract is only shown in one hemisphere here. The depicted tracts are right STN‐IFG (blue), right STN‐preSMA (green) and left IFG‐preSMA (pink), imposed over the mean b0 image.

Once we had the refined tractogram from *tck2connectome*, we used *connectome2tck* to identify the streamlines between each pair of the three ROIs, that is, the streamlines between IFG‐preSMA, STN‐IFG and STN‐preSMA in each hemisphere. An example of three of the six tracts for one participant can be found in Figure 3. A more detailed visualisation of these tracts (including axial, sagittal and coronal views across multiple participants) is available in the Supporting Information.

#### 2.2.4 Estimation of Generalised FA and ADC

Rather than the conventional estimation methods for FA (using the diffusion tensor model), we estimated generalised FA (GFA) as per Tuch (2004):

(1) $$\mathrm{GFA}=\sqrt{\frac{n\sum_{i=1}^{n}{\left(\Psi_{u_{i}}-\bar{\Psi }\right)}^{2}}{\left(n-1\right)\sum_{i=1}^{n}{\Psi_{u_{i}}}^{2}}}$$

This approach makes use of the white matter FODs (generated in 2.2.3), with ψ given as the FOD coefficients. This poses several advantages over tensor estimation methods. Traditional tensor‐based FA estimation assumes a single dominant diffusion direction per voxel, which is computationally simple but may inadequately represent the complexity of white matter microstructure. Given that standard DWI sequences have a resolution of 1–2 mm^3^ while axon diameters range from 1 to 20 μm, a single voxel often contains thousands of axons forming multiple tracts with different orientations. Up to 90% of voxels contain multiple fibre populations (Jeurissen et al. 2013; Schilling et al. 2021), meaning that diffusion estimates based on a single dominant direction can oversimplify the underlying anatomy. Particularly, these approaches tend to favour the identification of major connectivity pathways, limiting reconstruction of more minor pathways (Behrens et al. 2007; Dell'Acqua and Tournier 2019; Volz et al. 2018) and making estimates unreliable, particularly in highly‐crossing regions (frequently referred to as the crossing fibre problem: Alexander et al. 2001; Behrens et al. 2003). Instead, the FOD‐based approach used for GFA preserves multidirectional anisotropy information, enabling a more accurate representation of microscopic fibre anisotropy, particularly in regions with pronounced curvature or extensive crossing fibres (Tan et al. 2015; Tuch 2004). It should be interpreted in the same manner as standard FA (i.e., it quantifies the extent to which water movement is anisotropic in that voxel).

ADC was calculated for each of the three *b* values, and these values were averaged to get a voxelwise, whole‐brain estimation of ADC per participant (Moreau et al. 2018):

(2) $$\mathrm{ADC}=\frac{1}{3}\left[-\frac{1}{700}\ln\left(\frac{\left\langle b700\right\rangle }{\left\langle b0\right\rangle }\right)-\frac{1}{1000}\ln\left(\frac{\left\langle b1000\right\rangle }{\left\langle b0\right\rangle }\right)-\frac{1}{1600}\ln\left(\frac{\left\langle b1600\right\rangle }{\left\langle b0\right\rangle }\right)\right]$$

### 2.3 Quantitative MRI (QMRI)

#### 2.3.1 Data Acquisition

The quantitative images were acquired at the Spinoza Centre for Neuroimaging with a Philips 7 T Achieva MRI scanner with a 32‐channel, phased‐array coil. T_1_, T_2_* and QSM contrasts were obtained using an MP2RAGEME (multiecho magnetisation‐prepared rapid gradient echo) sequence (Caan et al. 2019) with 0.7 mm isotropic resolution. MP2RAGEME is an extension of the MP2RAGE sequence (Marques et al. 2010), and acquires two rapid gradient echo images (GRE1 and GRE2) in the sagittal plane after a 180° inversion pulse and excitation pulses with inversion times of TI_1_ = 670 ms and TI_2_ = 3675.4 ms. A multiecho readout was added to the second inversion at four echo times (TE_1_ = 3 ms, TE_2,1–4_ = 3, 11.5, 19, 28.5 ms). Other scan parameters: FA_GRE1,GRE2_ = (4°, 4°), TR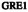 = 6.2 ms, TR_GRE2_ = 31 ms, TR_MPRAGEME_ = 6778 ms, acceleration factor SENSE_PA_ = 2, FOV = 205 × 205 × 164 mm, acquisition matrix = 292 × 290, bandwidth = 404.9 MHz, reconstructed voxel size = 0.67 × 0.67 × 0.7 mm, TFE (turbo factor) = 150 resulting in 176 shots. Total acquisition time = 19.53 min.

#### 2.3.2 Iron and Myelin Approximation

The procedure for estimating iron and myelin from this sample has been reported in full elsewhere (Miletić et al. 2022). In brief, preprocessing and the reconstruction of quantitative values was implemented in Python using Nighres (Huntenburg et al. 2018). Before reconstruction, the raw images were skull‐stripped and denoised using LCPCA (Bazin et al. 2019). Voxelwise values of iron and myelin were estimated for each participant using previously established models based on R1, R2* and quantitative susceptibility mapping (QSM) metrics, as described in Miletić et al. (2022):

(3) $$\begin{array}{c} \text{Iron}=0.24\times R2^{*}+98.28\times \mathrm{QSM}-3.83\\ \text{Myelin}=0.11\times R2^{*}+31.87\times R1-7.99 \end{array}$$

These estimations yielded whole‐brain maps with iron and myelin estimates per voxel for each participant. From here, we applied the masks for each of the ROIs and for the tracts of interest generated in 2.2.3, resulting in estimations of iron and myelin concentration in those regions and tracts for each participant. The overarching procedure is depicted in Figure 1.

### 2.4 Data Analysis

We investigated age‐related regional and network changes in the stopping network across four measures: iron, myelin, GFA and ADC. We first assessed age‐related alterations in iron and myelin in the IFG, preSMA and STN (regional changes), and then in iron, myelin, GFA and ADC in the IFG–preSMA, STN–IFG and STN–preSMA (network changes).

In each instance, we fit a series of polynomial regression models with participant age as a linear, quadratic, and/or cubic term. All models were fit with OLS, implemented using the Python package *statsmodels* (Seabold and Perktold 2010). Given the varying number of parameters in our candidate models, we compared the relative model fits using Bayesian information criterion (BIC), which quantifies the trade‐off between goodness‐of‐fit and model complexity (i.e., the number of free parameters in a model; Schwarz 1978). In each case, the model with the lowest BIC was taken as the winning model. For each winning model, we then identified influential datapoints using Cook's distance, with a threshold cutoff of 4/*n* (≈0.08; Rawlings et al. 1998). Individual datapoints that exceeded this threshold were removed. We then refit the data to each of the candidate models and re‐selected the winning model, again using BIC for model comparison.

We quantified the relationship between age and our measures of interest using two complementary approaches. First, we tested whether iron and myelin mediated the associations between age and GFA/ADC using mediation analyses. Second, we computed partial correlations among iron, myelin, ADC and GFA, controlling for age. Finally, to examine the relationships between anatomical changes in different regions, we assessed whether iron and myelin levels in specific regions mediated the associations between age and iron/myelin levels in the tracts.

## 3 Results

### 3.1 Averaging Between Hemispheres and Outlier Analysis

Student *t*‐tests were used to assess if there were any significant differences between hemispheres for any of the outcome variables for any region or tract. All *t*‐tests were nonsignificant (all p > 400). We therefore collapsed the data across hemispheres to reduce the number of fitted models and increase statistical power. The results of all *t*‐tests can be found in the Supporting Informations.

Outliers were identified using Cook's distance. After identifying each winning model, we removed influential datapoints using a threshold cutoff of 4/*n* (Rawlings et al. 1998) and refitted all candidate models on the data, excluding these datapoints, and reselecting the winning model. This removed an average of 2.67 datapoints per model (5.44% of total datapoints). Exclusions for each model can be found in the analysis code (https://osf.io/hnz79/).

### 3.2 Regional Changes (IFG, preSMA, STN)

We fit a series of polynomial regressions to assess age‐related changes in each of the individual regions of interest (IFG, preSMA, STN) for iron and myelin, with participant age as a linear, quadratic, and/or cubic term, using BIC for model comparison. The six model candidates can be found in the Supporting Information. Model comparison results for all candidate models (including BIC values) can be found at https://osf.io/hnz79/.

Parameterised winning models with corresponding BIC are shown in Table 1. Winning models showed linear age‐related changes in myelin for all three ROIs. Age‐related changes in iron were found to be linear for the IFG and preSMA, and quadratic for the STN.

**TABLE 1:** Age‐related changes in iron and myelin in the IFG, preSMA and STN.

Region	Measure	Winning model	BIC
IFG	Iron	γ=3.858+0.015x	91
	Myelin	γ=7.549−0.009x	115
preSMA	Iron	γ=4.510−0.010x	99
	Myelin	γ=8.046+0.031x	204
STN	Iron	$\gamma =6.732+0.371x-0.004x^{2}$	257
	Myelin	γ=15.353−0.011x	156

*Note:* Parameterised winning models showing age‐related changes in iron and myelin in the regions of interest. γ denotes the outcome variable (iron or myelin); *x* denotes age. Winning models were selected based on BIC.

### 3.3 Network Changes (IFG‐preSMA, STN‐IFG, STN‐preSMA)

#### 3.3.1 Age‐Related Changes in Iron, Myelin, GFA and ADC

We first assessed age‐related changes in the stopping network for the biophysical (iron and myelin) and diffusion measures (GFA and ADC). As in 3.2, we fit a series of polynomial regression models with participant age as a linear, quadratic, and/or cubic term, using BIC for model comparison. See Supporting Information for all candidate models. Model comparison results for all candidate models (including BIC values) can be found at https://osf.io/hnz79/. The parameterised winning models are described below and reported in Table 2 with their corresponding BIC.

**TABLE 2:** Age‐related changes in the stopping network.

Measure	Tract	Winning model	BIC
Myelin	IFG‐preSMA	γ=11.856+0.009x	115
	STN‐IFG	$\gamma =11.103+0.094x-0.001x^{2}$	100
	STN‐preSMA	$\gamma =11.961+0.099x-0.001x^{2}$	94
Iron	IFG‐preSMA	$\gamma =3.409+0.071x-0.001x^{2}$	79
	STN‐IFG	γ=5.159+0.019x	79
	STN‐preSMA	γ=4.812+0.011x	76
ADC	IFG‐preSMA	$\gamma =\left(7.96\times 10^{-4}\right)-\left(3.71\times 10^{-6}\right)x+\left(5.26\times 10^{-8}\right)x^{2}$	−798
	STN‐IFG	$\gamma =\left(7.51\times 10^{-4}\right)-\left(4.69\times 10^{-6}\right)x+\left(6.38\times 10^{-8}\right)x^{2}$	−793
	STN‐preSMA	$\gamma =\left(7.23\times 10^{-4}\right)-\left(3.41\times 10^{-6}\right)x+\left(4.67\times 10^{-8}\right)x^{2}$	−802
GFA	IFG‐preSMA	$\gamma =0.830-\left(3.90\times 10^{-5}\right)x$	−798
	STN‐IFG	$\gamma =0.851+\left(6.03\times 10^{-5}\right)x$	−793
	STN‐preSMA	$\gamma =0.859+\left(8.84\times 10^{-5}\right)x$	−802

*Note:* Parameterised winning models showing age‐related anatomical changes in the tracts of interest. γ denotes the outcome variable (iron, myelin, ADC or GFA); *x* denotes age. Note the coefficients are small for ADC and FA due to the small scale of these metrics.

##### 3.3.1.1 Iron and Myelin

The winning models showed linear age‐related increases in iron in the STN‐IFG and STN‐preSMA tracts, and quadratic changes in the IFG‐preSMA tract. The winning models showed quadratic changes in myelin in the STN‐IFG and STN‐preSMA tracts, and linear changes in the IFG‐preSMA tract.

##### 3.3.1.2 ADC and GFA

The winnings models identified quadratic changes in ADC for all three tracts of interest and linear changes for GFA. The coefficients for these models are particularly small due to their scale; normal white matter ADC ranges from 0.60 − 1.05 × 10^−3^mm^2^/s (Sener 2001), while GFA is a ratio, that is, values are between 0 and 1 (Figley et al. 2022). Note that some of the models showed small changes in BIC (e.g., BIC difference < 6), indicating there was not meaningful improvement in the model fit while accounting for model complexity (Raftery 1995). These models are interpreted with caution. All candidate models can be found in https://osf.io/hnz79/.

**FIGURE 4:**
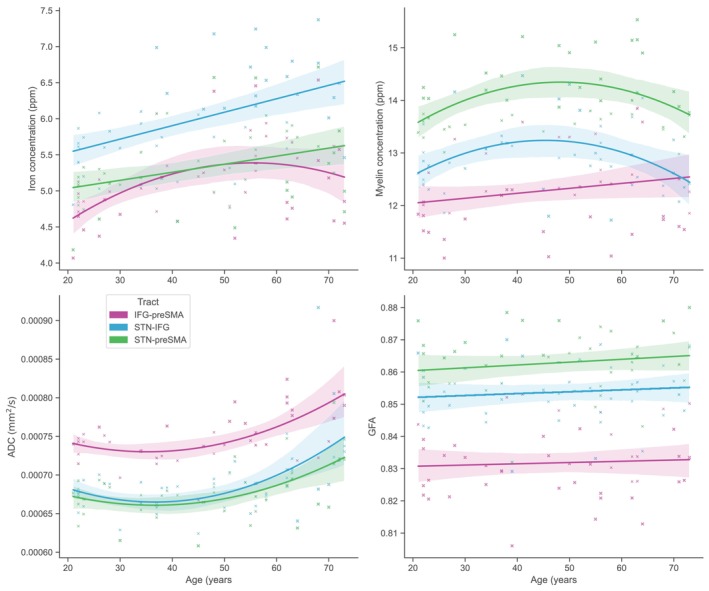
Age‐related changes in the IFG‐preSMA, STN‐IFG and STN‐preSMA tracts in iron (top left), myelin (top right), apparent diffusion coefficient (ADC; bottom left) and generalised fractional anisotropy (GFA; bottom right). Note that some models had relatively small (i.e., < 6) improvement in BIC, indicating that their fits should be interpreted with caution. Error bars depict confidence interval of the regression line.

Figure 4 shows the winning models for iron, myelin, ADC and GFA.

#### 3.3.2 Mediation and Partial Correlation Analyses

To further investigate the relationships between these variables, we conducted mediation analyses to assess whether iron and myelin influenced the association between age and diffusion measures in each tract of interest. Neither iron nor myelin significantly mediated the relationship between age and diffusion values (*p* > 0.872 for all tracts), suggesting that the associations between age and diffusion measures are independent of iron and myelin levels.

Next, we examined whether iron and myelin levels in specific regions mediated the relationship between age and those measures in the corresponding tracts (e.g., whether iron in the preSMA mediated the relationship between age and iron in the preSMA‐IFG tract). After correcting for multiple comparisons, we found that iron in the IFG significantly mediated the relationship between age and iron levels in both the IFG‐preSMA and STN‐IFG tracts (*p* = 0.003 and *p* = 0.005 respectively). No other significant mediation effects were observed for iron or myelin in any other regions (all *p* > 0.360). Estimates and significance tests for direct, indirect and total effects for each mediation analysis can be found in the Supporting Information.

We used partial correlations to investigate the relationship between the measures of interest (iron, myelin, ADC and GFA) per tract, with age given as a covariate in order to account for its effects. There was a weak to moderate negative correlation between almost all variables, except for iron and myelin, where the relationship was negligible (IFG‐preSMA: *r* = 0.034, STN‐IFG: *r* = −0.032, STN‐preSMA: *r* = 0.006). The full partial correlation matrices are reported in the Supporting Information.

We also ran a series of linear models to test for the effects of sex and potential age‐by‐sex interactions. These analyses revealed no significant main effects or interaction effects of sex in any model across all regions and tracts (all *p* > 0.061).

## 4 Discussion

The STN, IFG and preSMA play distinct roles in action cancellation. Current models of response inhibition propose that these regions, among others, operate as a network, coordinating activity in order to successfully cancel or modify volitional movement. This inhibitory ability is compromised in older populations, with older adults being slower to cancel their movements. In the current study, we quantified anatomical changes that occur in the STN, IFG and preSMA and their connecting pathways by combining high‐resolution DWI and ultra‐high field qMRI in the same participants, quantifying changes using polynomial regression. To our knowledge, this is the first study to examine age‐related biophysical and structural changes in the stopping network.

Broadly, we identified substantial age‐related anatomical change at the network level, that is, in the tracts that connect these regions, with less evidence for localised change, that is, anatomical change in the regions themselves. Taken together, the results suggest that the behavioural changes in response inhibition efficacy and altered neural recruitment patterns may be the product of network‐level breakdown, or connectivity alterations.

### 4.1 Minimal Localised Changes (ROIs)

We found the age‐related changes in iron or myelin to be mostly linear for the regions of interest (IFG, preSMA, STN), although these changes were minimal. Age‐related changes in iron concentration have previously been observed in the STN, although not always (for a review, see Madden and Merenstein 2023). It should be noted that estimation methods for iron vary. Here, we used a combination of QSM and R2* to estimate iron; previous work has often used QSM or R2* individually as proxies for iron (see, e.g., Betts et al. 2016; Burgetova et al. 2021; Keuken et al. 2017). The sample size of the current study was limited due to the availability of DWI data, and we have previously observed age‐related iron accumulation in the STN in larger samples (Miletić et al. 2022). Further, much previous work has focused on clinical samples and, particularly, identified iron accumulation patterns in clinical samples late in the disease course. Iron accumulation in the STN has been correlated with severe motor symptoms in late‐stage Parkinson's disease (PD) and has been hypothesised to be an important predictor of therapeutic and surgical outcomes (Brown et al. 2022; Huang et al. 2020). However, in early disease stages this association is less clear (Shin et al. 2018) and in healthy ageing contexts, the age‐related changes can be inconsistent (Madden and Merenstein 2023). Collectively, these findings suggest that local iron change may exhibit subtle variations, and the relationship between local anatomical changes and functional or behavioural change may be more difficult to describe, especially compared to broader network changes (see below, 4.2).

Turning to the cortex, to our knowledge there are no studies that have specifically examined age‐related changes in iron in the cortex in nonclinical populations. However, Burgetova et al. (2021) did identify age‐related changes in magnetic susceptibility in broader premotor and frontal regions, with these susceptibility changes assumed to be influenced by iron concentration. Varied broader cortical changes in some qMRI metrics in older adult populations have also been observed (Gracien et al. 2017; Seiler et al. 2020), including in prefrontal areas (Gozdas et al. 2021), but again, these are not particularly region‐specific. Although there are some limitations to our approach (see 4.5), this highlights the difficulty in capturing specific anatomical change even in relatively large cortical areas.

### 4.2 Age‐Related Changes in the Stopping Network

We observed quadratic and linear increases in iron in the tracts of interest (IFG‐preSMA, STN‐IFG and STN‐preSMA), quadratic and linear changes in myelin and quadratic increases in ADC. GFA showed a linear increase across the lifespan, although this change was minimal (see Figure 4). Further, our mediation analysis found very few indirect effects (see the Supporting Information), suggesting that the observed anatomical changes do not operate in a simple linear cascade. This interpretation is reinforced by the fact that the preferred models describing age‐related change were frequently nonlinear. These results imply that changes in iron, myelin and diffusivity may reflect relatively independent ageing processes, each contributing in distinct ways to the broader decline in inhibitory control.

#### 4.2.1 Iron

Iron is increasingly recognised as a significant factor in both diseased and healthy ageing contexts. While it is an essential component of neural functioning, iron is also an active oxidiser. Excessive iron accumulation is thought to be a factor in tissue degeneration, interrupting normal cellular functioning and causing degradation of cellular components such as mitochondria, lipids and proteins (Daugherty and Raz 2015; Hagemeier et al. 2012). Its accumulation has also been proposed to impact other neurophysiological measures, such as neural oscillatory patterns in the beta frequency band (Lin et al. 2023). Lin and colleagues propose that in a PD context, observed alterations in beta oscillatory activity are caused by excessive iron accumulation. This accumulation causes dopaminergic cell death in critical motor‐related regions, affecting subcortical activity mediated by acetylcholine and γ‐aminobutyric acid (GABA) and causing alterations in the intricate inhibitory circuitry of the subcortex. This ultimately leads to hyperactivation of the STN and the observed increase in beta oscillations. Beta oscillations have also been tightly coupled with stopping behaviour (Wessel 2020), with beta band coherence between frontal and motor regions (including the preSMA and IFG) correlated with stopping efficacy (Ding et al. 2023). GABAergic activity has also been linked to response inhibition in both younger and older adults, and older adults tend to show a reduction of GABAergic levels and activity (Faßbender et al. 2021; Hermans et al. 2019; Quetscher et al. 2015; Verstraelen et al. 2021). Here, we have shown patterns of iron accumulation in the stopping network across the adult lifespan. Tentatively, this accumulation may be a critical component of the neural breakdown that occurs in this network in older age, which ultimately leads to the aforementioned observed neural and behavioural changes, including alterations to beta oscillatory activity and GABergic activity, and SSRT slowing.

#### 4.2.2 Myelin

Like iron, myelin is essential for healthy brain function, speeding signal conduction and facilitating longer‐range signal transmission (Radtke et al. 2007; Williamson and Lyons 2018; Yeatman et al. 2014). Changes in myelin are associated with typical healthy ageing, with a loss of myelin sheaths and myelinated nerve fibres affecting the functioning of neural circuits and contributing to normal cognitive decline (Peters 2009; Zhang et al. 2021). Altered demyelination patterns have also been observed in a number of neurodegenerative conditions such as AD and multiple sclerosis (Bartzokis 2011; Grussu et al. 2017; Radtke et al. 2007). These demyelination patterns have been proposed to be a critical element of the neural breakdown that occurs in AD (incidentally, stimulated by iron accumulation; see Bartzokis 2011; Bartzokis et al. 2007; Khattar et al. 2021).

Previous work has identified broad quadratic decreases in myelin with older age, similar to those observed in the current study (Buyanova and Arsalidou 2021; Khattar et al. 2021; Miletić et al. 2022). Note that just like in the case of iron, different MRI metrics have been used as proxies for myelin. For example, Yeatman et al. (2014) used R1, Khattar et al. (2021) used myelin water fraction, and Grydeland et al. (2019) used the T1/T2 ratio. Whilst these estimates have been associated with myelination, they have also been demonstrated to be impacted by other factors (e.g., the T1/T2 ratio is more strongly associated with axon density than myelin density; Grydeland et al. 2019). Comparison of findings should, therefore, be made with due caution. Here (using a combination of R1 and R2*), we observed some patterns of demyelination in cortical–subcortical tracts. Both the IFG and the preSMA, and their connections to the STN, are thought to be critical in action cancellation and motor control (forming the hyperdirect and indirect pathways: Aron et al. 2014; Bingham et al. 2023; Diesburg and Wessel 2021; Kemp et al. 2024; Munakata et al. 2011). Action cancellation itself, which is thought to be realised via these two pathways, is specifically compromised in older adult populations. More proactive or attentional elements of stopping performance are relatively well‐preserved in older age (Bloemendaal et al. 2016; Hermans et al. 2019; Kleerekooper et al. 2016). Speculatively, the decrease in myelination observed in the STN‐cortical tracts (STN‐preSMA and STN‐IFG) may represent some neuroanatomical changes that underpin these behavioural changes.

#### 4.2.3 ADC and FA

We observed marked quadratic increases in the stopping network in ADC, but minimal changes in GFA (see Figure 4). With respect to response inhibition networks, work to date has examined the relationship between response inhibition networks and FA, rather than GFA, but these findings have been highly variable. For example, Madsen et al. (2020) found FA correlated with SSRT in the preSMA only (i.e., not in the IFG or in any subcortical regions), whereas Coxon et al. (2012) found this association in the IFG. Boen et al. (2022) found an association between stopping accuracy and FA in an IFG subcomponent^1^‐preSMA tract only (other regions of interest included the caudate, putamen, insula and STN). These estimations of FA have largely been done using a tensor estimation method with relatively simple protocols, which has a number of limitations. An extensive comparison of estimation techniques is beyond the scope of this paper, but briefly, these methods assume that diffusivity in each voxel can be described in a single direction. This creates problems in instances of crossing fibres (which may occur in up to 90% of all voxels: Jeurissen et al. 2013; Schilling et al. 2021) and favours the identification of major connectivity pathways, limiting reconstruction of more minor pathways (Dell'Acqua and Tournier 2019; Volz et al. 2018).

Conversely, GFA is useful for estimating diffusivity in more complex tissue environments, that is, in regions where there are many crossing fibres (Glenn et al. 2015; Sun et al. 2023). Although like FA, GFA does correlate negatively with age (Porcu et al. 2021), previous work has found these changes to be highly specific from region to region, meaning generalised statements about age‐associated changes should be considered with caution (Teipel et al. 2014). Moreover, changes to white matter structure may have varying impacts on these metrics, particularly FA. They quantify relative diffusivity, meaning that if degradation to white matter causes uniform changes to diffusivity, FA can be largely unchanged despite gross changes in the white matter (Figley et al. 2022). Additionally, in tensor estimation approaches, only the diffusivity in the major direction is quantified. If there are increases in white matter density in one of the nonmajor directions, this will lower the anisotropy in that voxel, which will paradoxically lower FA, when there has actually been an *increase* in white matter density (Figley et al. 2022). This indicates that while FA is sensitive to anatomical changes, its interpretation in terms of specific causes remains challenging (Jones and Cercignani 2010). Instead, metrics which assess net diffusion, that is, not diffusion in a particular direction, may provide more sensitive estimations.

Converse to the GFA results, for ADC (a measure of net diffusion: Helenius et al. 2002), we found consistent age‐related increases in all tracts of interest. In young adults, mean diffusivity has been found to positively correlate with SSRT in STN‐IFG and STN‐preSMA tracts (Rae et al. 2015), indicating that white matter integrity is intrinsic to action cancellation efficacy. Here, we observed an age‐associated increase in ADC, which may be reflective of alterations in the underlying anatomy (importantly, these changes probably more reliably reflect anatomical degradation, as ADC has been demonstrated to be more sensitive to underlying anatomical changes than FA: Scheurer et al. 2011).

### 4.3 Models of Ageing

Theoretical frameworks of ageing such as STAC and CRUNCH postulate that functional recruitment changes observed in older adult populations are preceded by measurable anatomical changes. For example, the STAC model hypothesises that functional recruitment changes are preceded by anatomical change including brain volume reduction, connectivity/white matter changes, cortical thinning and dopamine depletion (Park and Reuter‐Lorenz 2009; Reuter‐Lorenz and Park 2014). These changes result in broader recruitment patterns, which compensate for the structural changes by recruiting a wider range of neural regions than was previously required. Here, we have shown, for the first time, evidence of complex anatomical changes specific to the stopping network, and suggest that these may precede or underpin the well‐documented functional and behavioural changes in response inhibition in older adult populations.

Crucially, the majority of these anatomical changes were observable in the tracts that connect the canonical stopping regions, and not the regions themselves, despite these regions showing blood‐oxygenation level dependent (BOLD) activity changes in older adult populations (Bloemendaal et al. 2016; Coxon et al. 2016). We suggest that it may be the connectivity changes that most crucially underpin these observed functional changes or at least are the most broadly measurable. This is a particularly salient point given the requirement for fast and efficacious transmission of information in subcortical–cortical pathways under response inhibition frameworks (Aron 2007; Diesburg and Wessel 2021; Narayanan et al. 2020), which may deteriorate in older age.

It should also be noted that variation in localised anatomical changes (i.e., in individual regions) is thought to be the cause of the substantial individual differences in age‐related cognitive change (Kang et al. 2022). Likewise, in the context of response inhibition, a range of anatomical measures has been found to modulate individual performance (see, e.g., Boy et al. 2010; Chowdhury et al. 2019; Coxon et al. 2016; Forstmann et al. 2012; King et al. 2012). More localised anatomical changes, for example, changes to the stopping regions themselves, were not particularly evident in the current study, but may be better elucidated with the use of behavioural data, where anatomical changes could be linked with individual cognitive or behavioural measures.

### 4.4 Myelin and Iron Do Not Mediate Diffusion Indices

DWI is a way of estimating the structure of the underlying white matter, based on the assumption that the movement in the white matter is more constrained than in the grey matter or cerebrospinal fluid. It is assumed that demyelination affects these measures, resulting in increases in diffusivity (i.e., the movement of water is less constrained). There are few studies that have correlated diffusion measures with non‐DWI approximations of myelin or ex vivo measures, particularly outside of a clinical context, meaning the extent to which these metrics do truly index myelin remains unclear (Arshad et al. 2016; Henriques et al. 2023; Lazari and Lipp 2021).

Here, we first identified relationships between age and the diffusion metrics, and then used a mediation approach to assess if iron and myelin were mediators of these relationships. We largely found no mediation effects (except for one instance, but the direct effect was still larger than the indirect effect, that is, the mediation effect was comparatively small). Given we are only examining a small number of tracts, these results should not be taken as representative of the whole brain. However, it does demonstrate that, particularly in an ageing context, diffusion metrics may not directly reflect myelination. This is perhaps unsurprising given the huge number of anatomical changes that take place in older age; while demyelination and iron accumulation are certainly a part of these, there are also broader anatomical changes including a loss of dendritic spines, decreases in brain volume and alterations of astrocytes and other microglia (Palmer and Ousman 2018; Yeatman et al. 2014), which more broadly affects the central nervous system and will also impact diffusivity. Thus, whilst diffusion metrics can be a highly useful metric for studying anatomical change, direct interpretations of the specific changes that they index should be made with caution.

### 4.5 Limitations

The present study has several limitations. First, the data in the present study are purely anatomical, that is, we have no behavioural data for the participants. Behavioural data were collected for this sample, but due to COVID‐19 restrictions, was gathered online, resulting in a small sample size and insufficient data quality for further analyses (the MRI data had been acquired prior to this time). Instead, we relied on brain‐behaviour relations reported in the literature to inform our interpretations. Future work should correlate these metrics (behavioural, qMRI and DWI) in the same sample to strengthen the findings, particularly given the emphasis on individual differences known to affect ageing and stop signal performance (Chowdhury et al. 2019; Forstmann et al. 2012; Isherwood et al. 2023).

Second, the iron and myelin metrics used in this sample are approximations. There is no direct way to measure iron and myelin content in vivo, but as discussed above, there are a number of different ways to approximate these metrics. In the current study, we used simplified biophysical linear models that estimated iron and myelin concentrations using a combination of qMRI contrast values. These models were selected from eight model candidates, with all possible combinations of R1, R2* and QSM parameters. Their predictability was verified using literature‐based concentrations (for details, see Miletić et al. 2022), but they are proxies nonetheless.

Third, imaging acquisition and analysis in older adults presents unique challenges. Age‐related anatomical changes are highly variable and can interfere with standard preprocessing pipelines, for example, by reducing the accuracy of brain parcellation (Alkemade et al. 2022; Bazin et al. 2020). Moreover, MRI values can be independently affected by age‐related alterations, which may confound results and obscure specific anatomical changes. For instance, white matter hyperintensities, commonly observed in older individuals, can affect diffusion metrics. These hyperintensities can be identified using fluid attenuated inversion recovery (FLAIR) scans (Tubi et al. 2020), but these were not part of the acquisition sequence in the present study. Additionally, we note that recent advances in diffusion MRI modelling, such as NODDI (neurite orientation dispersion and density imaging; Zhang et al. 2012) and SANDI (soma and neurite density imaging; Palombo et al. 2020), enable detailed in vivo estimations of microstructural properties, including grey matter changes. Unfortunately, these models require higher *b* values during acquisition than those used in the current dataset to produce reliable parameter estimates. As such, while their use was not feasible in this study, future studies may find them highly useful in estimating age‐related neuroanatomical change.

Finally, we chose to focus on three regions of interest in the present study. While these regions are well‐documented as being intrinsic to action cancellation (see e.g., Aron et al. 2014; Borgomaneri et al. 2020; Chen et al. 2020; Lee et al. 2016; Lofredi et al. 2021), there are many additional regions that have been associated with response inhibition, particularly in the subcortex. Theoretical models of stopping such as the PTC model postulate that response inhibition involves a complex interplay of cortical and subcortical regions including the substantia nigra, globus pallidus and striatum (Diesburg and Wessel 2021; Schmidt et al. 2013; Schmidt and Berke 2017), with experimental evidence also identifying the ventral tegmental area and thalamus (Isherwood et al. 2025; Tennyson et al. 2018). Due to difficulties imaging the subcortex (de Hollander et al. 2017; Miletić et al. 2020), the specific role of these regions remains undetermined; more research is required to disentangle their specific role in action cancellation. Future work could include a broader range of anatomical regions in order to more fully encompass the response inhibition‐associated regions.

## 5 Conclusion

A large body of imaging and brain stimulation research has identified the IFG, preSMA and STN as being intrinsic to action cancellation. These regions, among others, form a stopping ‘network’, and are thought to coordinate to realise action selection and cancellation through a series of subcortical–cortical pathways. The connectivity of these regions is known to be an important factor in action cancellation and stopping, with improved connectivity of these regions associated with increased performance in the stop‐signal task.

Here, we quantified the anatomical changes that occur in these regions across the lifespan and in the white matter pathways that connect them, combining high‐resolution DWI and ultra‐high field qMRI in the same sample. We found substantial changes in iron concentration in these tracts, increases in ADC, and some evidence for demyelination. Conversely, we found very little evidence for age‐related anatomical changes in the regions themselves. We propose that some of the functional changes observed in these regions in older adult populations (e.g., increased BOLD recruitment) are a reflection of alterations to the stopping network itself, that is, to connectivity changes, rather than to localised regional change.

## Author Contributions

**Sarah A. Kemp:** conceptualisation, methodology, software, validation, formal analysis, data curation, writing – original draft, writing – review and editing, Visualisation. **Pierre‐Louis Bazin:** conceptualisation, methodology, software, validation, investigation, data curation, writing – review and editing, supervision, project administration. **Steven Miletić:** investigation, data curation, software, validation, writing – review and editing. **Russell J. Boag:** validation, writing – review and editing. **Max C. Keuken:** conceptualisation, software, investigation, data curation, supervision. **Mark R. Hinder:** supervision. **Birte U. Forstmann:** conceptualisation, resources, writing – review and editing, supervision, project administration, funding acquisition.

## Conflicts of Interest

The authors declare no conflicts of interest.

## Supporting information


Data S1.

## Data Availability

Data and code availability statement: The preprocessed DWI and qMRI datasets have been previously published and are publicly available (Alkemade et al. 2020; Keuken et al. 2022). All code used to estimate the tractography, run analyses and generate figures can be found at https://osf.io/hnz79/. The data that support the findings of this study are available as part of the Amsterdam Ultra‐high field adult lifespan database (AHEAD) at https://doi.org/10.21942/uva.10007840.v1 and https://uvaauas.figshare.com/projects/A_high‐resolution_multi‐shell_3T_diffusion_magnetic_resonance_imaging_dataset_as_part_of_the_Amsterdam_Ultra‐high_field_adult_lifespan_database_AHEAD_/125377.

## Endnotes

^1^
IFG dorsal pars opercularis.

## References

[hbm70240-bib-0001] Alexander, A. L. , K. M. Hasan , M. Lazar , J. S. Tsuruda , and D. L. Parker . 2001. “Analysis of Partial Volume Effects in Diffusion‐Tensor MRI.” Magnetic Resonance in Medicine 45, no. 5: 770–780. 10.1002/mrm.1105. 11323803

[hbm70240-bib-0002] Alkemade, A. , M. J. Mulder , J. M. Groot , et al. 2020. “The Amsterdam Ultra‐High Field Adult Lifespan Database (AHEAD): A Freely Available Multimodal 7 Tesla Submillimeter Magnetic Resonance Imaging Database.” NeuroImage 221: 117200. 10.1016/j.neuroimage.2020.117200. 32745682

[hbm70240-bib-0003] Alkemade, A. , M. J. Mulder , A. C. Trutti , and B. U. Forstmann . 2022. “Manual Delineation Approaches for Direct Imaging of the Subcortex.” Brain Structure and Function 227, no. 1: 219–297. 10.1007/s00429-021-02400-x. 34714408 PMC8741717

[hbm70240-bib-0004] Aron, A. R. 2007. “The Neural Basis of Inhibition in Cognitive Control.” Neuroscientist 13, no. 3: 214–228. 10.1177/1073858407299288. 17519365

[hbm70240-bib-0005] Aron, A. R. , T. E. Behrens , S. Smith , M. J. Frank , and R. A. Poldrack . 2007. “Triangulating a Cognitive Control Network Using Diffusion‐Weighted Magnetic Resonance Imaging (MRI) and Functional MRI.” Journal of Neuroscience 27, no. 14: 3743–3752. 10.1523/JNEUROSCI.0519-07.2007. 17409238 PMC6672420

[hbm70240-bib-0006] Aron, A. R. , and R. A. Poldrack . 2006. “Cortical and Subcortical Contributions to Stop Signal Response Inhibition: Role of the Subthalamic Nucleus.” Journal of Neuroscience 26, no. 9: 2424–2433. 10.1523/JNEUROSCI.4682-05.2006. 16510720 PMC6793670

[hbm70240-bib-0007] Aron, A. R. , T. W. Robbins , and R. A. Poldrack . 2004. “Inhibition and the Right Inferior Frontal Cortex.” Trends in Cognitive Sciences 8, no. 4: 170–177. 10.1016/j.tics.2004.02.010. 15050513

[hbm70240-bib-0008] Aron, A. R. , T. W. Robbins , and R. A. Poldrack . 2014. “Inhibition and the Right Inferior Frontal Cortex: One Decade on.” Trends in Cognitive Sciences 18, no. 4: 177–185. 10.1016/j.tics.2013.12.003. 24440116

[hbm70240-bib-0009] Arshad, M. , J. A. Stanley , and N. Raz . 2016. “Adult Age Differences in Subcortical Myelin Content Are Consistent With Protracted Myelination and Unrelated to Diffusion Tensor Imaging Indices.” NeuroImage 143: 26–39. 10.1016/j.neuroimage.2016.08.047. 27561713 PMC5124541

[hbm70240-bib-0010] Bartzokis, G. 2011. “Alzheimer's Disease as Homeostatic Responses to Age‐Related Myelin Breakdown.” Neurobiology of Aging 32, no. 8: 1341–1371. 10.1016/j.neurobiolaging.2009.08.007. 19775776 PMC3128664

[hbm70240-bib-0011] Bartzokis, G. , T. A. Tishler , P. H. Lu , et al. 2007. “Brain Ferritin Iron May Influence Age‐ and Gender‐Related Risks of Neurodegeneration.” Neurobiology of Aging 28, no. 3: 414–423. 10.1016/j.neurobiolaging.2006.02.005. 16563566

[hbm70240-bib-0012] Bazin, P.‐L. , A. Alkemade , M. J. Mulder , A. G. Henry , and B. U. Forstmann . 2020. “Multi‐Contrast Anatomical Subcortical Structures Parcellation.” eLife 9: e59430. 10.7554/eLife.59430. 33325368 PMC7771958

[hbm70240-bib-0013] Bazin, P.‐L. , A. Alkemade , W. van der Zwaag , M. Caan , M. Mulder , and B. U. Forstmann . 2019. “Denoising High‐Field Multi‐Dimensional MRI With Local Complex PCA.” Frontiers in Neuroendocrine Science 13: 1066. 10.3389/fnins.2019.01066. PMC6794471 31649500

[hbm70240-bib-0014] Beck, D. , A.‐M. G. de Lange , I. I. Maximov , et al. 2021. “White Matter Microstructure Across the Adult Lifespan: A Mixed Longitudinal and Cross‐Sectional Study Using Advanced Diffusion Models and Brain‐Age Prediction.” NeuroImage 224: 117441. 10.1016/j.neuroimage.2020.117441. 33039618

[hbm70240-bib-0015] Bedard, A.‐C. , S. Nichols , J. Barbosa , R. Schachar , G. Logan , and R. Tannock . 2002. “The Development of Selective Inhibitory Control Across the Life Span.” Developmental Neuropsychology 21, no. 1: 93–111. 10.1207/S15326942DN2101_5. 12058837

[hbm70240-bib-0016] Behrens, T. E. J. , H. J. Berg , S. Jbabdi , M. F. S. Rushworth , and M. W. Woolrich . 2007. “Probabilistic Diffusion Tractography With Multiple Fibre Orientations: What Can We Gain?” NeuroImage 34, no. 1: 144–155. 10.1016/j.neuroimage.2006.09.018. 17070705 PMC7116582

[hbm70240-bib-0017] Behrens, T. E. J. , M. W. Woolrich , M. Jenkinson , et al. 2003. “Characterization and Propagation of Uncertainty in Diffusion‐Weighted MR Imaging.” Magnetic Resonance in Medicine 50, no. 5: 1077–1088. 10.1002/mrm.10609. 14587019

[hbm70240-bib-0018] Betts, M. J. , J. Acosta‐Cabronero , A. Cardenas‐Blanco , P. J. Nestor , and E. Düzel . 2016. “Highresolution Characterisation of the Aging Brain Using Simultaneous Quantitative Susceptibility Mapping (QSM) and R2* Measurements at 7T.” NeuroImage 138: 43–63. 10.1016/j.neuroimage.2016.05.024. 27181761

[hbm70240-bib-0019] Bingham, C. S. , M. V. Petersen , M. Parent , and C. C. McIntyre . 2023. “Evolving Characterization of the Human Hyperdirect Pathway.” Brain Structure and Function 228, no. 2: 353–365. 10.1007/s00429-023-02610-5. 36708394 PMC10716731

[hbm70240-bib-0020] Bissett, P. G. , and G. D. Logan . 2011. “Balancing Cognitive Demands: Control Adjustments in the Stop‐Signal Paradigm.” Journal of Experimental Psychology: Learning, Memory, and Cognition 37, no. 2: 392–404. 10.1037/a0021800. 21171806 PMC3064521

[hbm70240-bib-0021] Bloemendaal, M. , B. Zandbelt , J. Wegman , O. van de Rest , R. Cools , and E. Aarts . 2016. “Contrasting Neural Effects of Aging on Proactive and Reactive Response Inhibition.” Neurobiology of Aging 46: 96–106. 10.1016/j.neurobiolaging.2016.06.007. 27460154

[hbm70240-bib-0022] Boen, R. , L. Raud , and R. J. Huster . 2022. “Inhibitory Control and the Structural Parcelation of the Right Inferior Frontal Gyrus.” Frontiers in Human Neuroscience 16: 787079. 10.3389/fnhum.2022.787079. 35280211 PMC8907402

[hbm70240-bib-0023] Borgomaneri, S. , G. Serio , and S. Battaglia . 2020. “Please, Don't Do It! Fifteen Years of Progress of Non‐Invasive Brain Stimulation in Action Inhibition.” Cortex 132: 404–422. 10.1016/j.cortex.2020.09.002. 33045520

[hbm70240-bib-0024] Boy, F. , C. J. Evans , R. A. Edden , K. D. Singh , M. Husain , and P. Sumner . 2010. “Individual Differences in Subconscious Motor Control Predicted by GABA Concentration in SMA.” Current Biology 20, no. 19: 1779–1785. 10.1016/j.cub.2010.09.003. 20888227 PMC3128986

[hbm70240-bib-0025] Brown, G. , G. Du , E. Farace , et al. 2022. “Subcortical Iron Accumulation Pattern May Predict Neuropsychological Outcomes After Subthalamic Nucleus Deep Brain Stimulation: A Pilot Study.” Journal of Parkinson's Disease 12, no. 3: 851–863. 10.3233/JPD-212833. PMC9181238 34974437

[hbm70240-bib-0026] Burgetova, R. , P. Dusek , A. Burgetova , et al. 2021. “Age‐Related Magnetic Susceptibility Changes in Deep Grey Matter and Cerebral Cortex of Normal Young and Middle‐Aged Adults Depicted by Whole Brain Analysis.” Quantitative Imaging in Medicine and Surgery 11, no. 9: 3906–3919. 10.21037/qims-21-87. 34476177 PMC8339659

[hbm70240-bib-0027] Buyanova, I. S. , and M. Arsalidou . 2021. “Cerebral White Matter Myelination and Relations to Age, Gender, and Cognition: A Selective Review.” Frontiers in Human Neuroscience 15: 662031. 10.3389/fnhum.2021.662031. 34295229 PMC8290169

[hbm70240-bib-0028] Caan, M. W. A. , P.‐L. Bazin , J. P. Marques , G. de Hollander , S. O. Dumoulin , and W. van der Zwaag . 2019. “MP2RAGEME: T1, T2*, and QSM Mapping in One Sequence at 7 Tesla.” Human Brain Mapping 40, no. 6: 1786–1798. 10.1002/hbm.24490. 30549128 PMC6590660

[hbm70240-bib-0029] Cavanagh, J. F. , J. L. Sanguinetti , J. J. B. Allen , S. J. Sherman , and M. J. Frank . 2014. “The Subthalamic Nucleus Contributes to Post‐Error Slowing.” Journal of Cognitive Neuroscience 26, no. 11: 2637–2644. 10.1162/jocn_a_00659. 24800632

[hbm70240-bib-0030] Chen, W. , C. de Hemptinne , A. M. Miller , et al. 2020. “Prefrontal‐Subthalamic Hyperdirect Pathway Modulates Movement Inhibition in Humans.” Neuron 106, no. 4: 579–588.e3. 10.1016/j.neuron.2020.02.012. 32155442 PMC7274135

[hbm70240-bib-0031] Chowdhury, N. S. , E. J. Livesey , and J. A. Harris . 2019. “Individual Differences in Intracortical Inhibition During Behavioural Inhibition.” Neuropsychologia 124: 55–65. 10.1016/j.neuropsychologia.2019.01.008. 30654018

[hbm70240-bib-0032] Coudé, D. , A. Parent , and M. Parent . 2018. “Single‐Axon Tracing of the Corticosubthalamic Hyperdirect Pathway in Primates.” Brain Structure and Function 223, no. 9: 3959–3973. 10.1007/s00429-018-1726-x. 30109491

[hbm70240-bib-0033] Coxon, J. P. , D. J. Goble , I. Leunissen , A. Van Impe , N. Wenderoth , and S. P. Swinnen . 2016. “Functional Brain Activation Associated With Inhibitory Control Deficits in Older Adults.” Cerebral Cortex 26, no. 1: 12–22. 10.1093/cercor/bhu165. 25085883

[hbm70240-bib-0034] Coxon, J. P. , A. Van Impe , N. Wenderoth , and S. P. Swinnen . 2012. “Aging and Inhibitory Control of Action: Cortico‐Subthalamic Connection Strength Predicts Stopping Performance.” Journal of Neuroscience 32, no. 24: 8401–8412. 10.1523/JNEUROSCI.6360-11.2012. 22699920 PMC6703643

[hbm70240-bib-0035] Daugherty, A. M. , and N. Raz . 2015. “Appraising the Role of Iron in Brain Aging and Cognition: Promises and Limitations of MRI Methods.” Neuropsychology Review 25, no. 3: 272–287. 10.1007/s11065-015-9292-y. 26248580 PMC4565753

[hbm70240-bib-0036] de Hollander, G. , M. Keuken , W. van der Zwaag , B. Forstmann , and R. Trampel . 2017. “Comparing Functional MRI Protocols for Small, Iron‐Rich Basal Ganglia Nuclei Such as the Subthalamic Nucleus at 7 T and 3 T.” Human Brain Mapping 38, no. 6: 3226–3248. 10.1002/hbm.23586. 28345164 PMC6867009

[hbm70240-bib-0037] Dell'Acqua, F. , and J.‐D. Tournier . 2019. “Modelling White Matter With Spherical Deconvolution: How and Why?” NMR in Biomedicine 32, no. 4: e3945. 10.1002/nbm.3945. 30113753 PMC6585735

[hbm70240-bib-0038] Diesburg, D. A. , and J. R. Wessel . 2021. “The Pause‐Then‐Cancel Model of Human Action‐Stopping: Theoretical Considerations and Empirical Evidence.” Neuroscience & Biobehavioral Reviews 129: 17–34. 10.1016/j.neubiorev.2021.07.019. 34293402 PMC8574992

[hbm70240-bib-0039] Ding, Q. , T. Lin , G. Cai , et al. 2023. “Individual Differences in Beta‐Band Oscillations Predict Motor‐Inhibitory Control.” Frontiers in Neuroscience 17: 1131862. 10.3389/fnins.2023.1131862. 36937674 PMC10014589

[hbm70240-bib-0040] Eagle, D. M. , C. Baunez , D. M. Hutcheson , O. Lehmann , A. P. Shah , and T. W. Robbins . 2008. “Stop‐Signal Reaction‐Time Task Performance: Role of Prefrontal Cortex and Subthalamic Nucleus.” Cerebral Cortex 18, no. 1: 178–188. 10.1093/cercor/bhm044. 17517682

[hbm70240-bib-0041] Faßbender, K. , K. Bey , J. V. Lippold , B. Aslan , R. Hurlemann , and U. Ettinger . 2021. “GABAergic Modulation of Performance in Response Inhibition and Interference Control Tasks.” Journal of Psychopharmacology 35, no. 12: 1496–1509. 10.1177/02698811211032440. 34278874

[hbm70240-bib-0042] Figley, C. R. , M. N. Uddin , K. Wong , J. Kornelsen , J. Puig , and T. D. Figley . 2022. “Potential Pitfalls of Using Fractional Anisotropy, Axial Diffusivity, and Radial Diffusivity as Biomarkers of Cerebral White Matter Microstructure.” Frontiers in Neuroscience 15: 799576. 10.3389/fnins.2021.799576. 35095400 PMC8795606

[hbm70240-bib-0043] Forstmann, B. U. , M. C. Keuken , S. Jahfari , et al. 2012. “Cortico‐Subthalamic White Matter Tract Strength Predicts Interindividual Efficacy in Stopping a Motor Response.” NeuroImage 60, no. 1: 370–375. 10.1016/j.neuroimage.2011.12.044. 22227131

[hbm70240-bib-0044] Frank, M. J. 2006. “Hold Your Horses: A Dynamic Computational Role for the Subthalamic Nucleus in Decision Making.” Neural Networks: The Official Journal of the International Neural Network Society 19, no. 8: 1120–1136. 10.1016/j.neunet.2006.03.006. 16945502

[hbm70240-bib-0045] Geerligs, L. , R. J. Renken , E. Saliasi , N. M. Maurits , and M. M. Lorist . 2015. “A Brain‐Wide Study of Age‐Related Changes in Functional Connectivity.” Cerebral Cortex 25, no. 7: 1987–1999. 10.1093/cercor/bhu012. 24532319

[hbm70240-bib-0046] Glenn, G. R. , J. A. Helpern , A. Tabesh , and J. H. Jensen . 2015. “Quantitative Assessment of Diffusional Kurtosis Anisotropy.” NMR in Biomedicine 28, no. 4: 448–459. 10.1002/nbm.3271. 25728763 PMC4378654

[hbm70240-bib-0047] Gozdas, E. , H. Fingerhut , H. Wu , et al. 2021. “Quantitative Measurement of Macromolecular Tissue Properties in White and Gray Matter in Healthy Aging and Amnestic MCI.” NeuroImage 237: 118161. 10.1016/j.neuroimage.2021.118161. 34000394 PMC8285270

[hbm70240-bib-0048] Gracien, R.‐M. , L. Nürnberger , P. Hok , et al. 2017. “Evaluation of Brain Ageing: A Quantitative Longitudinal MRI Study Over 7 Years.” European Radiology 27, no. 4: 1568–1576. 10.1007/s00330-016-4485-1. 27379992

[hbm70240-bib-0049] Graybiel, A. M. 2000. “The Basal Ganglia.” Current Biology 10, no. 14: R509–R511. 10.1016/S0960-9822(00)00593-5. 10899013

[hbm70240-bib-0050] Grier, M. D. 2020. “Estimating Brain Connectivity With Diffusion‐Weighted MRI: Promise and Peril.” Biological Psychiatry: Cognitive Neuroscience and Neuroimaging 5, no. 9: 846–854. 10.1016/j.bpsc.2020.04.009. 32513555 PMC7483308

[hbm70240-bib-0051] Grussu, F. , T. Schneider , C. Tur , et al. 2017. “Neurite Dispersion: A New Marker of Multiple Sclerosis Spinal Cord Pathology?” Annals of Clinical and Translational Neurology 4, no. 9: 663–679. 10.1002/acn3.445. 28904988 PMC5590517

[hbm70240-bib-0052] Grydeland, H. , P. E. Vértes , F. Váša , et al. 2019. “Waves of Maturation and Senescence in Micro‐Structural MRI Markers of Human Cortical Myelination Over the Lifespan.” Cerebral Cortex 29, no. 3: 1369–1381. 10.1093/cercor/bhy330. 30590439 PMC6373687

[hbm70240-bib-0053] Hagemeier, J. , J. J. Geurts , and R. Zivadinov . 2012. “Brain Iron Accumulation in Aging and Neurodegenerative Disorders.” Expert Review of Neurotherapeutics 12, no. 12: 1467–1480. 10.1586/ern.12.128. 23237353

[hbm70240-bib-0054] Hagmann, P. , L. Cammoun , X. Gigandet , et al. 2008. “Mapping the Structural Core of Human Cerebral Cortex.” PLoS Biology 6, no. 7: e159. 10.1371/journal.pbio.0060159. 18597554 PMC2443193

[hbm70240-bib-0055] Healey, R. , M. Goldsworthy , S. Salomoni , et al. 2024. “Impaired Motor Inhibition During Perceptual Inhibition in Older, but Not Younger Adults: A Psychophysiological Study.” Scientific Reports 14, no. 1: 2023. 10.1038/s41598-024-52269-z. 38263414 PMC10805883

[hbm70240-bib-0056] Helenius, J. , L. Soinne , J. Perkiö , et al. 2002. “Diffusion‐Weighted MR Imaging in Normal Human Brains in Various Age Groups.” American Journal of Neuroradiology 23, no. 2: 194. https://www.ncbi.nlm.nih.gov/pmc/articles/PMC7975251/. 11847041 PMC7975251

[hbm70240-bib-0057] Henriques, R. N. , R. Henson , Cam‐CAN , and M. M. Correia . 2023. “Unique Information From Common Diffusion MRI Models About White‐Matter Differences Across the Human Adult Lifespan.” Imaging Neuroscience 1: 1–25. 10.1162/imag_a_00051. PMC12327084 40799710

[hbm70240-bib-0058] Hermans, L. , C. Maes , L. Pauwels , et al. 2019. “Age‐Related Alterations in the Modulation of Intracortical Inhibition During Stopping of Actions.” Aging 11, no. 2: 371–385. 10.18632/aging.101741. 30670675 PMC6366958

[hbm70240-bib-0059] Hsieh, S. , and Y.‐C. Lin . 2017. “Stopping Ability in Younger and Older Adults: Behavioral and Event‐Related Potential.” Cognitive, Affective, & Behavioral Neuroscience 17, no. 2: 348–363. 10.3758/s13415-016-0483-7. 27896714

[hbm70240-bib-0060] Hu, S. , J. S. Ide , H. H. Chao , et al. 2018. “Structural and Functional Cerebral Bases of Diminished Inhibitory Control During Healthy Aging.” Human Brain Mapping 39, no. 12: 5085–5096. 10.1002/hbm.24347. 30113124 PMC6287913

[hbm70240-bib-0061] Hu, S. , M. Job , S. Jenks , H. Chao , and C.‐S. Li . 2019. “Imaging the Effects of Age on Proactive Control in Healthy Adults.” Brain Imaging and Behavior 13, no. 6: 1526–1537. 10.1007/s11682-019-00103-w. 31011949 PMC6812594

[hbm70240-bib-0062] Huang, W. , R. Ogbuji , L. Zhou , L. Guo , Y. Wang , and B. H. Kopell . 2020. “Motoric Impairment Versus Iron Deposition Gradient in the Subthalamic Nucleus in Parkinson's Disease.” Journal of Neurosurgery 135, no. 1: 284–290. 10.3171/2020.5.JNS201163. 32764171

[hbm70240-bib-0063] Huntenburg, J. M. , C. J. Steele , and P.‐L. Bazin . 2018. “Nighres: Processing Tools for High‐Resolution Neuroimaging.” GigaScience 7, no. 7: giy082. 10.1093/gigascience/giy082. 29982501 PMC6065481

[hbm70240-bib-0064] Isherwood, S. J. S. , P. Bazin , S. Miletić , et al. 2023. “Investigating Intra‐Individual Networks of Response Inhibition and Interference Resolution Using 7T MRI.” NeuroImage 271: 119988. 10.1016/j.neuroimage.2023.119988. 36868392

[hbm70240-bib-0065] Isherwood, S. J. S. , S. A. Kemp , S. Miletić , N. Stevenson , P.‐L. Bazin , and B. U. Forstmann . 2025. “Multi‐Study fMRI Outlooks on Subcortical BOLD Responses in the Stop‐Signal Paradigm.” eLife 12: 4. 10.7554/eLife.88652.4. PMC11753779 39841120

[hbm70240-bib-0066] Isherwood, S. J. S. , M. C. Keuken , P. L. Bazin , and B. U. Forstmann . 2021. “Cortical and Subcortical Contributions to Interference Resolution and Inhibition—An fMRI ALE Meta‐Analysis.” Neuroscience & Biobehavioral Reviews 129: 245–260. 10.1016/j.neubiorev.2021.07.021. 34310977

[hbm70240-bib-0067] Jeurissen, B. , A. Leemans , J.‐D. Tournier , D. K. Jones , and J. Sijbers . 2013. “Investigating the Prevalence of Complex Fiber Configurations in White Matter Tissue With Diffusion Magnetic Resonance Imaging.” Human Brain Mapping 34, no. 11: 2747–2766. 10.1002/hbm.22099. 22611035 PMC6870534

[hbm70240-bib-0068] Jeurissen, B. , J.‐D. Tournier , T. Dhollander , A. Connelly , and J. Sijbers . 2014. “Multi‐Tissue Constrained Spherical Deconvolution for Improved Analysis of Multi‐Shell Diffusion MRI Data.” NeuroImage 103: 411–426. 10.1016/j.neuroimage.2014.07.061. 25109526

[hbm70240-bib-0069] Jones, D. K. , and M. Cercignani . 2010. “Twenty‐Five Pitfalls in the Analysis of Diffusion MRI Data.” NMR in Biomedicine 23, no. 7: 803–820. 10.1002/nbm.1543. 20886566

[hbm70240-bib-0070] Kang, W. , J. Wang , and A. Malvaso . 2022. “Inhibitory Control in Aging: The Compensation‐Related Utilization of Neural Circuits Hypothesis.” Frontiers in Aging Neuroscience 13: 771885. https://www.frontiersin.org/articles/10.3389/fnagi.2021.771885. 35967887 10.3389/fnagi.2021.771885 PMC9371469

[hbm70240-bib-0071] Kemp, S. A. , S. Salomoni , P.‐L. Bazin , L. Pash , R. J. S. George , and M. R. Hinder . 2024. “Cortical Contributions to Attentional Orienting and Response Cancellation in Action Stopping.” bioRxiv: 622650. 10.1101/2024.11.08.622650. PMC13548964 42704230

[hbm70240-bib-0072] Kennedy, K. M. , K. M. Rodrigue , G. N. Bischof , A. C. Hebrank , P. A. Reuter‐Lorenz , and D. C. Park . 2015. “Age Trajectories of Functional Activation Under Conditions of Low and High Processing Demands: An Adult Lifespan fMRI Study of the Aging Brain.” NeuroImage 104: 21–34. 10.1016/j.neuroimage.2014.09.056. 25284304 PMC4252495

[hbm70240-bib-0073] Keuken, M. C. , P.‐L. Bazin , K. Backhouse , et al. 2017. “Effects of Aging on T1, T2*, and QSM MRI Values in the Subcortex.” Brain Structure and Function 222, no. 6: 2487–2505. 10.1007/s00429-016-1352-4. 28168364 PMC5541117

[hbm70240-bib-0074] Keuken, M. C. , L. C. Liebrand , P.‐L. Bazin , et al. 2022. “A High‐Resolution Multi‐Shell 3T Diffusion Magnetic Resonance Imaging Dataset as Part of the Amsterdam Ultra‐High Field Adult Lifespan Database (AHEAD).” Data in Brief 42: 108086. 10.1016/j.dib.2022.108086. 35372652 PMC8971326

[hbm70240-bib-0075] Khattar, N. , C. Triebswetter , M. Kiely , et al. 2021. “Investigation of the Association Between Cerebral Iron Content and Myelin Content in Normative Aging Using Quantitative Magnetic Resonance Neuroimaging.” NeuroImage 239: 118267. 10.1016/j.neuroimage.2021.118267. 34139358 PMC8370037

[hbm70240-bib-0076] King, A. V. , J. Linke , A. Gass , et al. 2012. “Microstructure of a Three‐Way Anatomical Network Predicts Individual Differences in Response Inhibition: A Tractography Study.” NeuroImage 59, no. 2: 1949–1959. 10.1016/j.neuroimage.2011.09.008. 21939775

[hbm70240-bib-0077] Kleerekooper, I. , S. J. H. van Rooij , W. P. M. van den Wildenberg , M. de Leeuw , R. S. Kahn , and M. Vink . 2016. “The Effect of Aging on Fronto‐Striatal Reactive and Proactive Inhibitory Control.” NeuroImage 132: 51–58. 10.1016/j.neuroimage.2016.02.031. 26899783

[hbm70240-bib-0078] Lazari, A. , and I. Lipp . 2021. “Can MRI Measure Myelin? Systematic Review, Qualitative Assessment, and Meta‐Analysis of Studies Validating Microstructural Imaging With Myelin Histology.” NeuroImage 230: 117744. 10.1016/j.neuroimage.2021.117744. 33524576 PMC8063174

[hbm70240-bib-0079] Lebel, C. , M. Gee , R. Camicioli , M. Wieler , W. Martin , and C. Beaulieu . 2012. “Diffusion Tensor Imaging of White Matter Tract Evolution Over the Lifespan.” NeuroImage 60, no. 1: 340–352. 10.1016/j.neuroimage.2011.11.094. 22178809

[hbm70240-bib-0080] Lee, H. W. , M.‐S. Lu , C.‐Y. Chen , N. G. Muggleton , T.‐Y. Hsu , and C.‐H. Juan . 2016. “Roles of the Pre‐SMA and rIFG in Conditional Stopping Revealed by Transcranial Magnetic Stimulation.” Behavioural Brain Research 296: 459–467. 10.1016/j.bbr.2015.08.024. 26304720

[hbm70240-bib-0081] Lin, M. , G. Cai , Y. Li , et al. 2023. “Association Between Beta Oscillations From Subthalamic Nucleus and Quantitative Susceptibility Mapping in Deep Gray Matter Structures in Parkinson's Disease.” Brain Sciences 13, no. 1: 81. 10.3390/brainsci13010081. 36672062 PMC9857066

[hbm70240-bib-0082] Lofredi, R. , G. C. Auernig , F. Irmen , et al. 2021. “Subthalamic Stimulation Impairs Stopping of Ongoing Movements.” Brain 144, no. 1: 44–52. 10.1093/brain/awaa341. 33253351

[hbm70240-bib-0083] Madden, D. J. , and J. L. Merenstein . 2023. “Quantitative Susceptibility Mapping of Brain Iron in Healthy Aging and Cognition.” NeuroImage 282: 120401. 10.1016/j.neuroimage.2023.120401. 37802405 PMC10797559

[hbm70240-bib-0084] Madsen, K. S. , L. B. Johansen , W. K. Thompson , H. R. Siebner , T. L. Jernigan , and W. F. C. Baaré . 2020. “Maturational Trajectories of White Matter Microstructure Underlying the Right Presupplementary Motor Area Reflect Individual Improvements in Motor Response Cancellation in Children and Adolescents.” NeuroImage 220: 117105. 10.1016/j.neuroimage.2020.117105. 32615252

[hbm70240-bib-0085] Marques, J. P. , T. Kober , G. Krueger , W. van der Zwaag , P.‐F. Van de Moortele , and R. Gruetter . 2010. “MP2RAGE, a Self Bias‐Field Corrected Sequence for Improved Segmentation and T1‐Mapping at High Field.” NeuroImage 49, no. 2: 1271–1281. 10.1016/j.neuroimage.2009.10.002. 19819338

[hbm70240-bib-0086] Miletić, S. , P.‐L. Bazin , S. J. S. Isherwood , M. C. Keuken , A. Alkemade , and B. U. Forstmann . 2022. “Charting Human Subcortical Maturation Across the Adult Lifespan With In Vivo 7 T MRI.” NeuroImage 249: 118872. 10.1016/j.neuroimage.2022.118872. 34999202

[hbm70240-bib-0087] Miletić, S. , P.‐L. Bazin , N. Weiskopf , W. van der Zwaag , B. U. Forstmann , and R. Trampel . 2020. “fMRI Protocol Optimization for Simultaneously Studying Small Subcortical and Cortical Areas at 7T.” NeuroImage 219: 116992. 10.1016/j.neuroimage.2020.116992. 32480037

[hbm70240-bib-0088] Moreau, B. , A. Iannessi , C. Hoog , and H. Beaumont . 2018. “How Reliable Are ADC Measurements? A Phantom and Clinical Study of Cervical Lymph Nodes.” European Radiology 28, no. 8: 3362–3371. 10.1007/s00330-017-5265-2. 29476218 PMC6028847

[hbm70240-bib-0089] Munakata, Y. , S. A. Herd , C. H. Chatham , B. E. Depue , M. T. Banich , and R. C. O'Reilly . 2011. “A Unified Framework for Inhibitory Control.” Trends in Cognitive Sciences 15, no. 10: 453–459. 10.1016/j.tics.2011.07.011. 21889391 PMC3189388

[hbm70240-bib-0090] Nambu, A. , H. Tokuno , and M. Takada . 2002. “Functional Significance of the Cortico–Subthalamo–Pallidal ‘Hyperdirect’ Pathway.” Neuroscience Research 43, no. 2: 111–117. 10.1016/S0168-0102(02)00027-5. 12067746

[hbm70240-bib-0091] Narayanan, N. S. , J. R. Wessel , and J. D. W. Greenlee . 2020. “The Fastest Way to Stop: Inhibitory Control and IFG‐STN Hyperdirect Connectivity.” Neuron 106, no. 4: 549–551. 10.1016/j.neuron.2020.04.017. 32437650 PMC8188636

[hbm70240-bib-0092] Neubert, F.‐X. , R. B. Mars , J. Sallet , and M. F. S. Rushworth . 2015. “Connectivity Reveals Relationship of Brain Areas for Reward‐Guided Learning and Decision Making in Human and Monkey Frontal Cortex.” Proceedings of the National Academy of Sciences 112, no. 20: E2695–E2704. 10.1073/pnas.1410767112. PMC4443352 25947150

[hbm70240-bib-0093] Neubert, F.‐X. , R. B. Mars , A. G. Thomas , J. Sallet , and M. F. Rushworth . 2014. “Comparison of Human Ventral Frontal Cortex Areas for Cognitive Control and Language With Areas in Monkey Frontal Cortex.” Neuron 81, no. 3: 700–713. 10.1016/j.neuron.2013.11.012. 24485097

[hbm70240-bib-0094] Nikitenko, T. , N. Chowdhury , R. Puri , and J. L. He . 2020. “Response Inhibition in Humans: A Whistle Stop Review.” Journal of Neurophysiology 123, no. 3: 861–864. 10.1152/jn.00572.2019. 31664878

[hbm70240-bib-0095] Nougaret, S. , J. Meffre , Y. Duclos , E. Breysse , and Y. Pelloux . 2013. “First Evidence of a Hyperdirect Prefrontal Pathway in the Primate: Precise Organization for New Insights on Subthalamic Nucleus Functions.” Frontiers in Computational Neuroscience 7: 135. 10.3389/fncom.2013.00135. 24133443 PMC3794292

[hbm70240-bib-0096] O'Donnell, L. J. , and C.‐F. Westin . 2011. “An Introduction to Diffusion Tensor Image Analysis.” Neurosurgery Clinics of North America 22, no. 2: 185. 10.1016/j.nec.2010.12.004. 21435570 PMC3163395

[hbm70240-bib-0097] Palmer, A. L. , and S. S. Ousman . 2018. “Astrocytes and Aging.” Frontiers in Aging Neuroscience 10: 00337. 10.3389/fnagi.2018.00337. PMC6212515 30416441

[hbm70240-bib-0098] Palombo, M. , A. Ianus , M. Guerreri , et al. 2020. “SANDI: A Compartment‐Based Model for Non‐Invasive Apparent Soma and Neurite Imaging by Diffusion MRI.” NeuroImage 215: 116835. 10.1016/j.neuroimage.2020.116835. 32289460 PMC8543044

[hbm70240-bib-0099] Park, D. C. , and P. Reuter‐Lorenz . 2009. “The Adaptive Brain: Aging and Neurocognitive Scaffolding.” Annual Review of Psychology 60: 173–196. 10.1146/annurev.psych.59.103006.093656. PMC3359129 19035823

[hbm70240-bib-0100] Peters, A. 2009. “The Effects of Normal Aging on Myelinated Nerve Fibers in Monkey Central Nervous System.” Frontiers in Neuroanatomy 3: 11. 10.3389/neuro.05.011.2009. 19636385 PMC2713738

[hbm70240-bib-0101] Porcu, M. , L. Cocco , J. Puig , et al. 2021. “Global Fractional Anisotropy: Effect on Resting‐State Neural Activity and Brain Networking in Healthy Participants.” Neuroscience 472: 103–115. 10.1016/j.neuroscience.2021.07.021. 34364954

[hbm70240-bib-0102] Puri, R. , T. Nikitenko , and S. A. Kemp . 2018. “Using Transcranial Magnetic Stimulation to Investigate the Neural Mechanisms of Inhibitory Control.” Journal of Neurophysiology 120, no. 4: 1587–1590. 10.1152/jn.00366.2018. 30020843

[hbm70240-bib-0103] Quetscher, C. , A. Yildiz , S. Dharmadhikari , et al. 2015. “Striatal GABA‐MRS Predicts Response Inhibition Performance and Its Cortical Electrophysiological Correlates.” Brain Structure and Function 220, no. 6: 3555–3564. 10.1007/s00429-014-0873-y. 25156575 PMC4447607

[hbm70240-bib-0104] Radtke, C. , M. Spies , M. Sasaki , P. Vogt , and J. D. Kocsis . 2007. “Demyelinating Diseases and Potential Repair Strategies.” International Journal of Developmental Neuroscience 25, no. 3: 149–153. 10.1016/j.ijdevneu.2007.02.002. 17408905 PMC2692731

[hbm70240-bib-0105] Rae, C. , L. Hughes , M. Anderson , and J. Rowe . 2015. “The Prefrontal Cortex Achieves Inhibitory Control by Facilitating Subcortical Motor Pathway Connectivity.” Journal of Neuroscience 35, no. 2: 786–794. 10.1523/JNEUROSCI.3093-13.2015. 25589771 PMC4293423

[hbm70240-bib-0106] Raftery, A. E. 1995. “Bayesian Model Selection in Social Research.” Sociological Methodology 25: 111–163. 10.2307/271063.

[hbm70240-bib-0107] Rawlings, J. O. , S. G. Pantula , and D. A. Dickey , eds. 1998. Applied Regression Analysis. Springer‐Verlag. 10.1007/b98890.

[hbm70240-bib-0108] Reuter‐Lorenz, P. A. , and D. C. Park . 2014. “How Does It STAC Up? Revisiting the Scaffolding Theory of Aging and Cognition.” Neuropsychology Review 24, no. 3: 355–370. 10.1007/s11065-014-9270-9. 25143069 PMC4150993

[hbm70240-bib-0109] Rey‐Mermet, A. , and M. Gade . 2018. “Inhibition in Aging: What Is Preserved? What Declines? A Meta‐Analysis.” Psychonomic Bulletin & Review 25, no. 5: 1695–1716. 10.3758/s13423-017-1384-7. 29019064

[hbm70240-bib-0110] Rocha, G. S. , M. A. M. Freire , A. M. Britto , et al. 2023. “Basal Ganglia for Beginners: The Basic Concepts You Need to Know and Their Role in Movement Control.” Frontiers in Systems Neuroscience 17: 1242929. 10.3389/fnsys.2023.1242929. 37600831 PMC10435282

[hbm70240-bib-0111] Scheurer, E. , K.‐O. Lovblad , R. Kreis , et al. 2011. “Forensic Application of Postmortem Diffusion‐Weighted and Diffusion Tensor MR Imaging of the Human Brain In Situ.” American Journal of Neuroradiology 32, no. 8: 1518–1524. 10.3174/ajnr.A2508. 21659482 PMC7964364

[hbm70240-bib-0112] Schilling, K. G. , C. M. W. Tax , F. Rheault , et al. 2021. “Prevalence of White Matter Pathways Coming Into a Single White Matter Voxel Orientation: The Bottleneck Issue in Tractography.” Human Brain Mapping 43, no. 4: 1196–1213. 10.1002/hbm.25697. 34921473 PMC8837578

[hbm70240-bib-0113] Schmidt, R. , and J. D. Berke . 2017. “A Pause‐Then‐Cancel Model of Stopping: Evidence From Basal Ganglia Neurophysiology.” Philosophical Transactions of the Royal Society of London. Series B, Biological Sciences 372, no. 1718: 20160202. 10.1098/rstb.2016.0202. 28242736 PMC5332861

[hbm70240-bib-0114] Schmidt, R. , D. K. Leventhal , N. Mallet , F. Chen , and J. D. Berke . 2013. “Canceling Actions Involves a Race Between Basal Ganglia Pathways.” Nature Neuroscience 16, no. 8: 1118–1124. 10.1038/nn.3456. 23852117 PMC3733500

[hbm70240-bib-0115] Schmidt, S. , R. M. Cichy , A. Kraft , J. Brocke , K. Irlbacher , and S. A. Brandt . 2009. “An Initial Transient‐State and Reliable Measures of Corticospinal Excitability in TMS Studies.” Clinical Neurophysiology 120, no. 5: 987–993. 10.1016/j.clinph.2009.02.164. 19359215

[hbm70240-bib-0116] Schroll, H. , and F. H. Hamker . 2013. “Computational Models of Basal‐Ganglia Pathway Functions: Focus on Functional Neuroanatomy.” Frontiers in Systems Neuroscience 7: 122. 10.3389/fnsys.2013.00122. 24416002 PMC3874581

[hbm70240-bib-0117] Schwarz, G. 1978. “Estimating the Dimension of a Model.” Annals of Statistics 6, no. 2: 461–464. 10.1214/aos/1176344136.

[hbm70240-bib-0118] Seabold, S. , and J. Perktold . 2010. “Statsmodels: Econometric and Statistical Modeling With Python.” SciPy 7, no. 1: 92–96. 10.25080/Majora-92bf1922-011.

[hbm70240-bib-0119] Sebastian, A. , C. Baldermann , B. Feige , et al. 2013. “Differential Effects of Age on Subcomponents of Response Inhibition.” Neurobiology of Aging 34, no. 9: 2183–2193. 10.1016/j.neurobiolaging.2013.03.013. 23591131

[hbm70240-bib-0120] Sebastian, A. , M. F. Pohl , S. Klöppel , et al. 2013. “Disentangling Common and Specific Neural Subprocesses of Response Inhibition.” NeuroImage 64: 601–615. 10.1016/j.neuroimage.2012.09.020. 22986077

[hbm70240-bib-0121] Seiler, A. , S. Schöngrundner , B. Stock , et al. 2020. “Cortical Aging – New Insights With Multiparametric Quantitative MRI.” Aging (Albany NY) 12, no. 16: 16195–16210. 10.18632/aging.103629. 32852283 PMC7485732

[hbm70240-bib-0122] Sener, R. N. 2001. “Diffusion MRI: Apparent Diffusion Coefficient (ADC) Values in the Normal Brain and a Classification of Brain Disorders Based on ADC Values.” Computerized Medical Imaging and Graphics 25, no. 4: 299–326. 10.1016/S0895-6111(00)00083-5. 11356324

[hbm70240-bib-0123] Shin, C. , S. Lee , J.‐Y. Lee , J. H. Rhim , and S.‐W. Park . 2018. “Non‐Motor Symptom Burdens Are Not Associated With Iron Accumulation in Early Parkinson's Disease: A Quantitative Susceptibility Mapping Study.” Journal of Korean Medical Science 33, no. 13: e96. 10.3346/jkms.2018.33.e96. 29573246 PMC5865060

[hbm70240-bib-0124] Singh, M. , I. Fuelscher , J. He , V. Anderson , T. J. Silk , and C. Hyde . 2021. “Inter‐Individual Performance Differences in the Stop‐Signal Task Are Associated With Fibre‐Specific Microstructure of the Fronto‐Basal‐Ganglia Circuit in Healthy Children.” Cortex 142: 283–295. 10.1016/j.cortex.2021.06.002. 34315068

[hbm70240-bib-0125] Smith, R. E. , J.‐D. Tournier , F. Calamante , and A. Connelly . 2015. “SIFT2: Enabling Dense Quantitative Assessment of Brain White Matter Connectivity Using Streamlines Tractography.” NeuroImage 119: 338–351. 10.1016/j.neuroimage.2015.06.092. 26163802

[hbm70240-bib-0126] Smith, S. M. , M. Jenkinson , M. W. Woolrich , et al. 2004. “Advances in Functional and Structural MR Image Analysis and Implementation as FSL.” NeuroImage 23: S208–S219. 10.1016/j.neuroimage.2004.07.051. 15501092

[hbm70240-bib-0127] Smittenaar, P. , M. Guitart‐Masip , A. Lutti , and R. J. Dolan . 2013. “Preparing for Selective Inhibition Within Frontostriatal Loops.” Journal of Neuroscience 33, no. 46: 18087–18097. 10.1523/JNEUROSCI.2167-13.2013. 24227719 PMC3828462

[hbm70240-bib-0128] Sullivan, E. V. , and A. Pfefferbaum . 2006. “Diffusion Tensor Imaging and Aging.” Neuroscience and Biobehavioral Reviews 30, no. 6: 749–761. 10.1016/j.neubiorev.2006.06.002. 16887187

[hbm70240-bib-0129] Sun, F. , Y. Huang , J. Wang , W. Hong , and Z. Zhao . 2023. “Research Progress in Diffusion Spectrum Imaging.” Brain Sciences 13, no. 10: 1497. 10.3390/brainsci13101497. 37891866 PMC10605731

[hbm70240-bib-0130] Tan, E. T. , L. Marinelli , J. I. Sperl , M. I. Menzel , and C. J. Hardy . 2015. “Multi‐Directional Anisotropy From Diffusion Orientation Distribution Functions.” Journal of Magnetic Resonance Imaging 41, no. 3: 841–850. 10.1002/jmri.24589. 24753055

[hbm70240-bib-0131] Tardif, C. L. , C. J. Gauthier , C. J. Steele , et al. 2016. “Advanced MRI Techniques to Improve Our Understanding of Experienceinduced Neuroplasticity.” NeuroImage 131: 55–72. 10.1016/j.neuroimage.2015.08.047. 26318050

[hbm70240-bib-0132] Teipel, S. J. , M. Lerche , I. Kilimann , et al. 2014. “Decline of Fiber Tract Integrity Over the Adult Age Range: A Diffusion Spectrum Imaging Study.” Journal of Magnetic Resonance Imaging 40, no. 2: 348–359. 10.1002/jmri.24420. 24923796

[hbm70240-bib-0133] Tennyson, S. S. , A. T. Brockett , N. W. Hricz , D. W. Bryden , and M. R. Roesch . 2018. “Firing of Putative Dopamine Neurons in Ventral Tegmental Area Is Modulated by Probability of Success During Performance of a Stop‐Change Task.” eNeuro 5, no. 2: 182018. 10.1523/ENEURO.0007-18.2018. PMC5909181 29687078

[hbm70240-bib-0134] Tournier, J.‐D. , F. Calamante , and A. Connelly . 2010. “Improved Probabilistic Streamlines Tractography by 2nd Order Integration Over Fibre Orientation Distributions.” In Proceedings of the International Society for Magnetic Resonance in Medicine. Wiley.

[hbm70240-bib-0135] Tournier, J.‐D. , F. Calamante , D. G. Gadian , and A. Connelly . 2004. “Direct Estimation of the Fiber Orientation Density Function From Diffusion‐Weighted MRI Data Using Spherical Deconvolution.” NeuroImage 23, no. 3: 1176–1185. 10.1016/j.neuroimage.2004.07.037. 15528117

[hbm70240-bib-0136] Tournier, J.‐D. , R. Smith , D. Raffelt , et al. 2019. “MRtrix3: A Fast, Flexible and Open Software Framework for Medical Image Processing and Visualisation.” NeuroImage 202: 116137. 10.1016/j.neuroimage.2019.116137. 31473352

[hbm70240-bib-0137] Tsvetanov, K. , Z. Ye , L. Hughes , et al. 2018. “Activity and Connectivity Differences Underlying Inhibitory Control Across the Adult Life Span.” Journal of Neuroscience 38, no. 36: 7887–7900. 10.1523/JNEUROSCI.2919-17.2018. 30049889 PMC6125816

[hbm70240-bib-0138] Tubi, M. A. , F. W. Feingold , D. Kothapalli , et al. 2020. “White Matter Hyperintensities and Their Relationship to Cognition: Effects of Segmentation Algorithm.” NeuroImage 206: 116327. 10.1016/j.neuroimage.2019.116327. 31682983 PMC6981030

[hbm70240-bib-0139] Tuch, D. S. 2004. “Q‐Ball Imaging.” Magnetic Resonance in Medicine 52, no. 6: 1358–1372. 10.1002/mrm.20279. 15562495

[hbm70240-bib-0140] Van De Laar, M. C. , W. P. van den Wildenberg , G. J. van Boxtel , and M. W. van der Molen . 2011. “Lifespan Changes in Global and Selective Stopping and Performance Adjustments.” Frontiers in Psychology 2: 00357. 10.3389/fpsyg.2011.00357. PMC3238363 22180746

[hbm70240-bib-0141] Verbruggen, F. , A. R. Aron , G. P. Band , et al. 2019. “A Consensus Guide to Capturing the Ability to Inhibit Actions and Impulsive Behaviors in the Stop‐Signal Task.” eLife 8: e46323. 10.7554/eLife.46323. 31033438 PMC6533084

[hbm70240-bib-0142] Verstraelen, S. , K. Cuypers , C. Maes , et al. 2021. “Neurophysiological Modulations in the (Pre)motormotor Network Underlying Age‐Related Increases in Reaction Time and the Role of GABA Levels – A Bimodal TMS‐MRS Study.” NeuroImage 243: 118500. 10.1016/j.neuroimage.2021.118500. 34428570 PMC8547554

[hbm70240-bib-0143] Volz, L. J. , M. Cieslak , and S. T. Grafton . 2018. “A Probabilistic Atlas of Fiber Crossings for Variability Reduction of Anisotropy Measures.” Brain Structure and Function 223, no. 2: 635–651. 10.1007/s00429-017-1508-x. 28905121

[hbm70240-bib-0144] Weiskopf, N. , S. Mohammadi , A. Lutti , and M. F. Callaghan . 2015. “Advances in MRI‐Based Computational Neuroanatomy: From Morphometry to In‐Vivo Histology.” Current Opinion in Neurology 28, no. 4: 313–322. 10.1097/WCO.0000000000000222. 26132532

[hbm70240-bib-0145] Wessel, J. R. 2020. “Beta‐Bursts Reveal the Trial‐To‐Trial Dynamics of Movement Initiation and Cancellation.” Journal of Neuroscience 40, no. 2: 411–423. 10.1523/JNEUROSCI.1887-19.2019. 31748375 PMC6948942

[hbm70240-bib-0146] Wessel, J. R. , and A. R. Aron . 2017. “On the Globality of Motor Suppression: Unexpected Events and Their Influence on Behavior and Cognition.” Neuron 93, no. 2: 259–280. 10.1016/j.neuron.2016.12.013. 28103476 PMC5260803

[hbm70240-bib-0147] Williams, B. R. , J. S. Ponesse , R. J. Schachar , G. D. Logan , and R. Tannock . 1999. “Development of Inhibitory Control Across the Life Span.” Developmental Psychology 35, no. 1: 205–213. 10.1037//0012-1649.35.1.205. 9923475

[hbm70240-bib-0148] Williamson, J. M. , and D. A. Lyons . 2018. “Myelin Dynamics Throughout Life: An Ever‐Changing Landscape?” Frontiers in Cellular Neuroscience 12: 424. 10.3389/fncel.2018.00424. 30510502 PMC6252314

[hbm70240-bib-0149] Xu, B. , M. Sandrini , W.‐T. Wang , et al. 2016. “PreSMA Stimulation Changes Task‐Free Functional Connectivity in the Fronto‐Basal‐Ganglia That Correlates With Response Inhibition Efficiency.” Human Brain Mapping 37, no. 9: 3236–3249. 10.1002/hbm.23236. 27144466 PMC4980157

[hbm70240-bib-0150] Yang, M.‐H. , Z.‐F. Yao , and S. Hsieh . 2019. “Multimodal Neuroimaging Analysis Reveals Ageassociated Common and Discrete Cognitive Control Constructs.” Human Brain Mapping 40, no. 9: 2639–2661. 10.1002/hbm.24550. 30779255 PMC6865786

[hbm70240-bib-0151] Yeatman, J. D. , B. A. Wandell , and A. A. Mezer . 2014. “Lifespan Maturation and Degeneration of Human Brain White Matter.” Nature Communications 5, no. 1: 4932. 10.1038/ncomms5932. PMC4238904 25230200

[hbm70240-bib-0152] Zandbelt, B. B. , M. Bloemendaal , J. M. Hoogendam , R. S. Kahn , and M. Vink . 2013. “Transcranial Magnetic Stimulation and Functional MRI Reveal Cortical and Subcortical Interactions During Stop‐Signal Response Inhibition.” Journal of Cognitive Neuroscience 25, no. 2: 157–174. 10.1162/jocn_a_00309. 23066733

[hbm70240-bib-0153] Zhang, F. , and S. Iwaki . 2020. “Correspondence Between Effective Connections in the Stop‐Signal Task and Microstructural Correlations.” Frontiers in Human Neuroscience 14: 279. 10.3389/fnhum.2020.00279. 32848664 PMC7396500

[hbm70240-bib-0154] Zhang, H. , T. Schneider , C. A. Wheeler‐Kingshott , and D. C. Alexander . 2012. “NODDI: Practical In Vivo Neurite Orientation Dispersion and Density Imaging of the Human Brain.” NeuroImage 61, no. 4: 1000–1016. 10.1016/j.neuroimage.2012.03.072. 22484410

[hbm70240-bib-0155] Zhang, X. , N. Huang , L. Xiao , F. Wang , and T. Li . 2021. “Replenishing the Aged Brains: Targeting Oligodendrocytes and Myelination?” Frontiers in Aging Neuroscience 13: 760200. 10.3389/fnagi.2021.760200. 34899272 PMC8656359

[hbm70240-bib-0156] Zhu, D. C. , R. T. Zacks , and J. M. Slade . 2010. “Brain Activation During Interference Resolution in Young and Older Adults: An fMRI Study.” NeuroImage 50, no. 2: 810–817. 10.1016/j.neuroimage.2009.12.087. 20045067 PMC2823923

[hbm70240-bib-0157] Zimmermann, J. , P. Ritter , K. Shen , S. Rothmeier , M. Schirner , and A. R. McIntosh . 2016. “Structural Architecture Supports Functional Organization in the Human Aging Brain at a Regionwise and Network Level.” Human Brain Mapping 37, no. 7: 2645–2661. 10.1002/hbm.23200. 27041212 PMC6867479

