# Supplementary material for: Neuroanatomical Changes in the Stopping Network Across the Adult Lifespan Assessed With Quantitative and Diffusion MRI
Source: Hum Brain Mapp. 2025 Jun 5;46(8):e70240. doi: 10.1002/hbm.70240 (PMC12138585; doi:10.1002/hbm.70240)
Supplement: Supplementary file 1 — Data S1. [file HBM-46-e70240-s001.docx]

**Neuroanatomical changes in the stopping network across the adult lifespan assessed with quantitative and diffusion MRI**

*Supplementary Materials*

**From 2.2.3: Tractography**

^
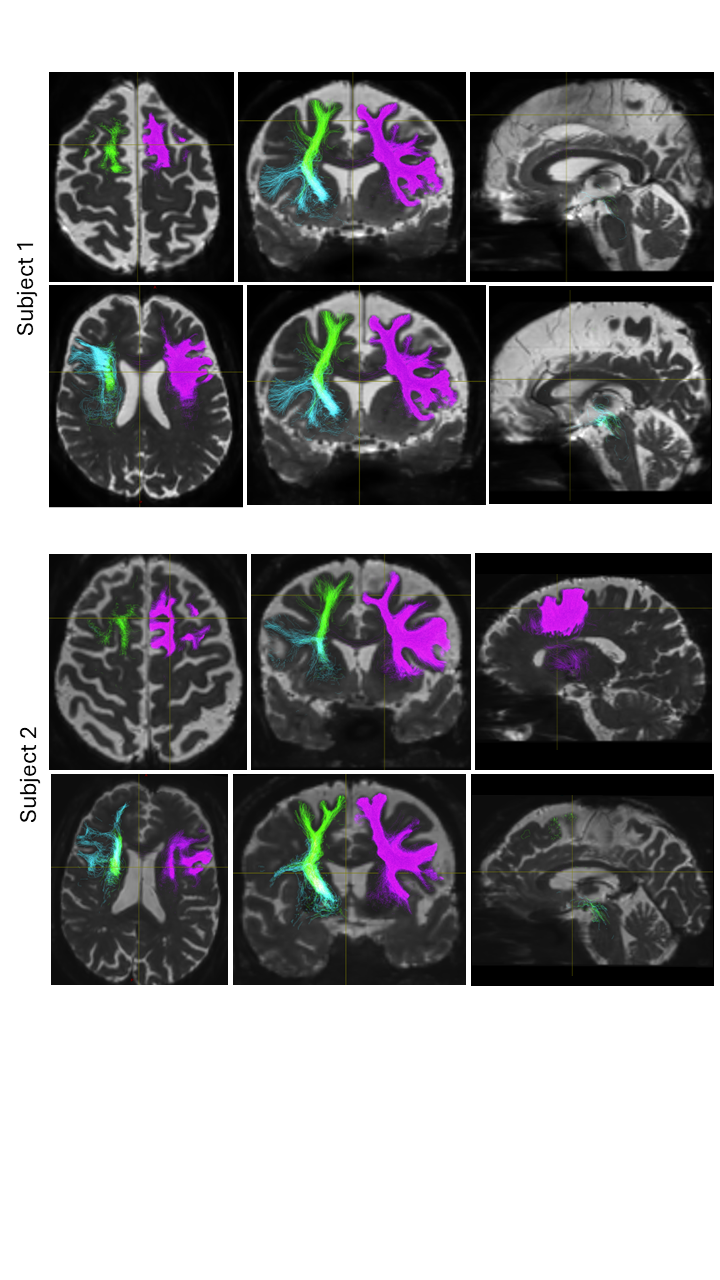
^

Additional view of the tracts of interest for two participants, given in axial, sagittal, and coronal view. Participants are aged in their 50s and 60s.

**From 3.1: *t-*test results**

Results of the Student *t*-test, assessing if there were any significant differences between hemispheres for any of the measures.

| Measure | Student’s *t* | *p*-value |
| --- | --- | --- |
| Regional iron | -0.35 | .730 |
| Tract iron | 0.69 | .493 |
| Regional myelin | -0.21 | .832 |
| Tract myelin | 0.13 | .897 |
| GFA | 0.83 | .408 |
| ADC | -0.69 | .494 |

Given all *p* > .05, we collapsed across hemispheres for all analyses to increase statistical power.

**From 3.2 and 3.3.1: Candidate models**

The following models were considered as candidates, with γ denoting the outcome variable (iron and myelin in 3.2; iron, myelin, GFA, and ADC in 3.3.1):

$$\gamma_{1}\equiv\beta_{1}Age$$

$$\gamma_{2}\equiv\beta_{1}Age +\beta_{2}{Age}^{2}$$

$$\gamma_{3}\equiv\beta_{1}Age +\beta_{2}{Age}^{2} +\beta_{3}{Age}^{3}$$

**From 3.3.2: Mediation and partial correlation analysis**

Results from the first mediation analysis, which assessed the influence of iron and myelin on the association between age and the diffusion measures (GFA and ADC) for each tract. The indirect effects (the average casual mediation effect: ACME, direct effects (average direct effects: ADE), and total effect (Total) are given. Statistically significant results are emphasised with *

| **DWI Metric** | **Tract** | **Mediator** | **Effect** | **Estimate** | ***p*-value** |
| --- | --- | --- | --- | --- | --- |
| GFA | IFG-preSMA | Iron | ACME | -2.93 × 10⁻⁷ | .988 |
|  |  |  | ADE | 9.13 × 10⁻⁵ | .352 |
|  |  |  | Total | 9.10 × 10⁻⁵ | .366 |
|  |  | Myelin | ACME | -3.54 × 10⁻⁷ | .950 |
|  |  |  | ADE | 6.68 × 10⁻⁵ | .458 |
|  |  |  | Total | 6.64 × 10⁻⁵ | .478 |
|  | STN-IFG | Iron | ACME | -8.14 × 10⁻⁷ | .964 |
|  |  |  | ADE | 1.85 × 10⁻⁴ | .016* |
|  |  |  | Total | 1.84 × 10⁻⁴ | .022* |
|  |  | Myelin | ACME | 9.36 × 10⁻⁸ | .984 |
|  |  |  | ADE | 5.85 × 10⁻⁵ | .482 |
|  |  |  | Total | 5.86 × 10⁻⁵ | .480 |
|  | STN-preSMA | Iron | ACME | 4.97 × 10⁻⁷ | .998 |
|  |  |  | ADE | 1.74 × 10⁻⁴ | .032* |
|  |  |  | Total | 1.74 × 10⁻⁴ | .030* |
|  |  | Myelin | ACME | -4.54 × 10⁻⁷ | .956 |
|  |  |  | ADE | 8.64 × 10⁻⁵ | .306 |
|  |  |  | Total | 8.59 × 10⁻⁵ | .304 |
| ADC | IFG-preSMA | Iron | ACME | -2.76 × 10⁻¹¹ | .918 |
|  |  |  | ADE | 1.52 × 10⁻⁶ | <.001* |
|  |  |  | Total | 1.52 × 10⁻⁶ | <.001* |
|  |  | Myelin | ACME | -3.09 × 10⁻¹² | .952 |
|  |  |  | ADE | 1.15 × 10⁻⁶ | <.001* |
|  |  |  | Total | 1.15 × 10⁻⁶ | <.001* |
|  |  |  | *Continued next page* | | |

| **DWI Metric** | **Tract** | **Mediator** | **Effect** | **Estimate** | ***p*-value** |
| --- | --- | --- | --- | --- | --- |
| ADC | STN-IFG | Iron | ACME | -2.04 × 10⁻¹¹ | .896 |
|  |  |  | ADE | 1.49 × 10⁻⁶ | <.001* |
|  |  |  | Total | 1.49 × 10⁻⁶ | <.001* |
|  |  | Myelin | ACME | -1.06 × 10⁻¹¹ | .964 |
|  |  |  | ADE | 1.18 × 10⁻⁶ | .004* |
|  |  |  | Total | 1.18 × 10⁻⁶ | .004* |
|  | STN-preSMA | Iron | ACME | -3.21 × 10⁻¹¹ | .872 |
|  |  |  | ADE | 1.46 × 10⁻⁶ | .002* |
|  |  |  | Total | 1.46 × 10⁻⁶ | .002* |
|  |  | Myelin | ACME | -1.07 × 10⁻¹¹ | .904 |
|  |  |  | ADE | 1.20 × 10⁻⁶ | <.001* |
|  |  |  | Total | 1.20 × 10⁻⁶ | <.001* |

Results from the second mediation analysis, which assessed whether iron and myelin levels in specific regions mediated the associations between age and iron/myelin levels in the tracts. The indirect effects (the average casual mediation effect: ACME, direct effects (average direct effects: ADE), and total effect (Total) are given. Statistically significant results (with an adjusted alpha level of *p* = .0125) are emphasised with *

| **Tract + measure** | **Mediator** | **Effect** | **Estimate** | ***p*-value** |
| --- | --- | --- | --- | --- |
| IFG-preSMA iron | IFG iron | ACME | 1.06 × 10⁻² | .003* |
|  |  | ADE | 2.38 × 10⁻³ | .503 |
|  |  | Total | 1.30 × 10⁻² | .010* |
|  | preSMA iron | ACME | -3.15 × 10⁻³ | .145 |
|  |  | ADE | 1.92 × 10⁻² | <.001* |
|  |  | Total | 1.60 × 10⁻² | .002* |
| IFG-preSMA myelin | IFG myelin | ACME | -6.45 × 10⁻³ | .203 |
|  |  | ADE | 1.54 × 10⁻² | .002* |
|  |  | Total | 8.93 × 10⁻³ | .185 |
|  | preSMA myelin | ACME | 6.70 × 10⁻³ | .035 |
|  |  | ADE | -2.98 × 10⁻³ | .560 |
|  |  | Total | 3.72 × 10⁻³ | .569 |
|  |  | *Continued next page* | | |

| **Tract + measure** | **Mediator** | **Effect** | **Estimate** | ***p*-value** |
| --- | --- | --- | --- | --- |
| STN-IFG iron | STN iron | ACME | -6.00 × 10⁻⁵ | .797 |
|  |  | ADE | 1.67 × 10⁻² | .002* |
|  |  | Total | 1.66 × 10⁻² | .002* |
|  | IFG iron | ACME | 8.95 × 10⁻³ | .005* |
|  |  | ADE | 8.50 × 10⁻³ | .031 |
|  |  | Total | 1.75 × 10⁻² | <.001* |
| STN-IFG myelin | STN myelin | ACME | -1.58 × 10⁻³ | .352 |
|  |  | ADE | -2.82 × 10⁻³ | .653 |
|  |  | Total | -4.40 × 10⁻³ | .491 |
|  | IFG myelin | ACME | -5.73 × 10⁻³ | .166 |
|  |  | ADE | 1.65 × 10⁻³ | .741 |
|  |  | Total | -4.07 × 10⁻³ | .520 |
| STN-preSMA iron | STN iron | ACME | -4.25 × 10⁻⁵ | .838 |
|  |  | ADE | 1.18 × 10⁻² | .018 |
|  |  | Total | 1.18 × 10⁻² | .017 |
|  | preSMA iron | ACME | -3.38 × 10⁻³ | .091 |
|  |  | ADE | 1.72 × 10⁻² | <.001* |
|  |  | Total | 1.38 × 10⁻² | .006* |
| STN-preSMA myelin | STN myelin | ACME | -1.74 × 10⁻³ | .360 |
|  |  | ADE | 1.77 × 10⁻³ | .779 |
|  |  | Total | 2.68 × 10⁻⁵ | .997 |
|  | preSMA myelin | ACME | 5.94 × 10⁻³ | .038 |
|  |  | ADE | -8.32 × 10⁻³ | .097 |
|  |  | Total | -2.38 × 10⁻³ | .702 |

Below are the heatmaps for the partial correlation analyses:

*
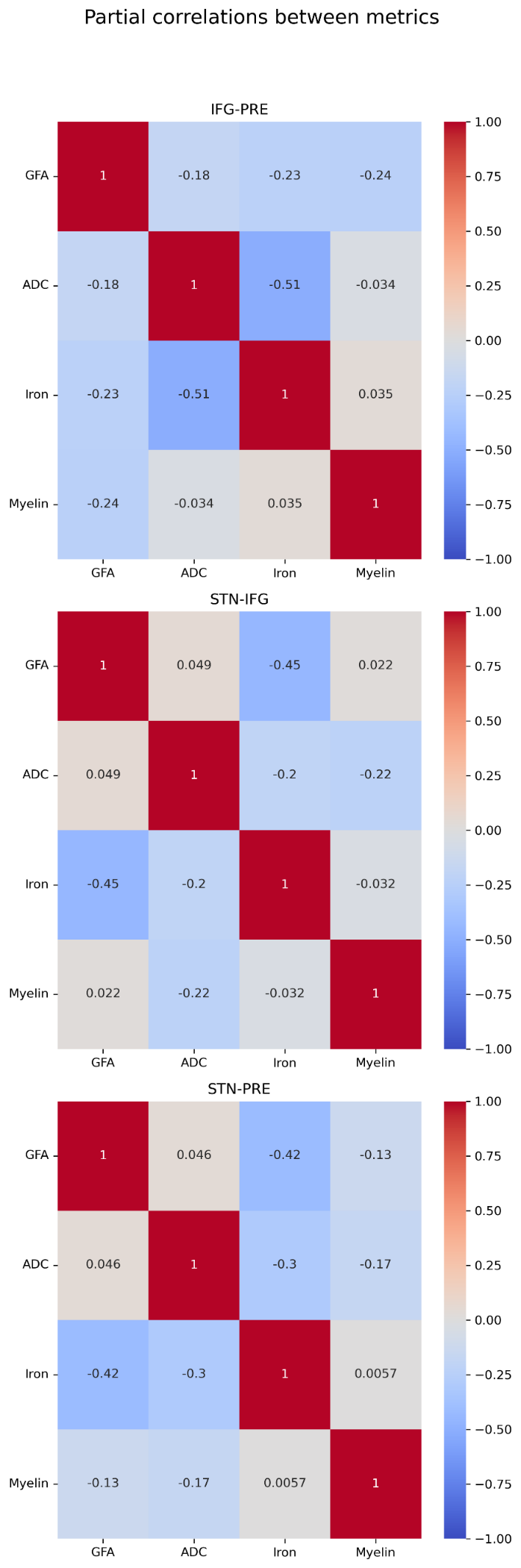
*

*
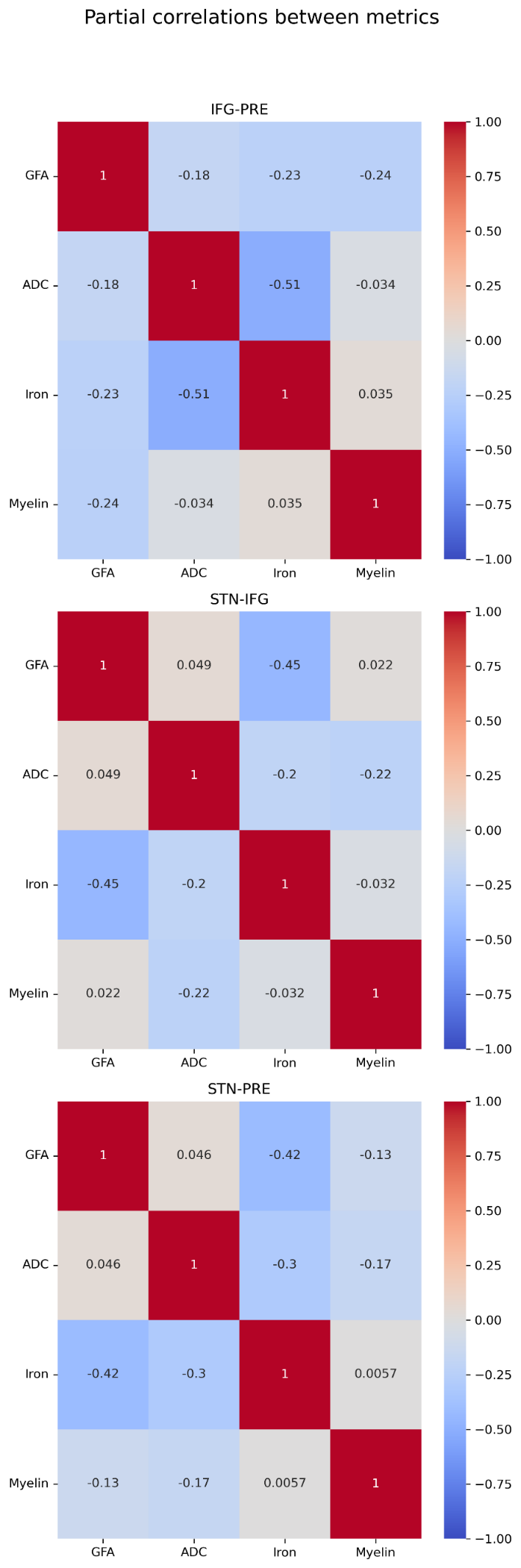
*
